# A DNA barcode library for ground beetles of Germany: the genus *Pterostichus* Bonelli, 1810 and allied taxa (Insecta, Coleoptera, Carabidae)

**DOI:** 10.3897/zookeys.980.55979

**Published:** 2020-10-28

**Authors:** Michael J. Raupach, Karsten Hannig, Jérome Morinière, Lars Hendrich

**Affiliations:** 1 Sektion Hemiptera, Bavarian State Collection of Zoology (SNSB – ZSM), Münchhausenstraße 21, 81247 München, Germany; 2 Bismarckstraße 5, 45731 Waltrop, Germany; 3 AIM – Advanced Identification Methods GmbH, Spinnereistraße 11, 04179 Leipzig; 4 Sektion Insecta varia, Bavarian State Collection of Zoology (SNSB – ZSM), Münchhausenstraße 21, 81247 München, Germany

**Keywords:** *
Abax
*, Central Europe, cytochrome *c* oxidase subunit I, German Barcode of Life, mitochondrial DNA, molecular specimen identification, *
Molops
*, *
Poecilus
*, *
Stomis
*

## Abstract

Species of the ground beetle genus *Pterostichus* Bonelli, 1810 are some of the most common carabids in Europe. This publication provides a first comprehensive DNA barcode library for this genus and allied taxa including *Abax* Bonelli, 1810, *Molops* Bonelli, 1810, *Poecilus* Bonelli, 1810, and *Stomis* Clairville, 1806 for Germany and Central Europe in general. DNA barcodes were analyzed from 609 individuals that represent 51 species, including sequences from previous studies as well as more than 198 newly generated sequences. The results showed a 1:1 correspondence between BIN and traditionally recognized species for 44 species (86%), whereas two (4%) species were characterized by two BINs. Three BINs were found for one species (2%), while one BIN for two species was revealed for two species pairs (8%). Low interspecific distances with maximum pairwise K2P values below 2.2% were found for four species pairs. Haplotype sharing was found for two closely related species pairs: *Pterostichus adstrictus* Eschscholtz, 1823/*Pterostichus oblongopunctatus* (Fabricius, 1787) and *Pterostichus nigrita* Paykull, 1790/*Pterostichus rhaeticus* Heer, 1837. In contrast to this, high intraspecific sequence divergences with values above 2.2% were shown for three species (*Molops piceus* (Panzer, 1793), *Pterostichus panzeri* (Panzer, 1805), *Pterostichus strenuus* (Panzer, 1793)). Summarizing the results, the present DNA barcode library does not only allow the identification of most of the analyzed species, but also provides valuable information for alpha-taxonomy as well as for ecological and evolutionary research. This library represents another step in building a comprehensive DNA barcode library of ground beetles as part of modern biodiversity research.

## Introduction

As part of the global International Barcode of Life initiative (IBoL; https://ibol.org), the German Barcode of Life initiative (GBoL; www.bolgermany.de) aims at capturing the genetic diversity of animals, fungi and plants of Germany using DNA barcodes in terms of modern biodiversity research (Hebert et al. 2003a, b). Despite the fact that various effects may limit the efficiency of a successful species identification, for example recent or ongoing hybridization events (e.g., Rougerie et al. 2012; Mutanen et al. 2016; Havemann et al. 2018), mitochondrial DNA-like sequences in the nucleus (numts) (e.g., Rogers and Griffiths-Jones 2012; Jordal and Kambestand 2014), or effects of *Wolbachia* infections (e.g., Smith et al. 2012; Klopfstein et al. 2016; Kolasa et al. 2018; Kajtoch et al. 2019), DNA barcoding has become the method of choice in terms of modern molecular species identification, including the identification of single specimens as well as metabarcoding of bulk samples (e.g., Casiraghi et al. 2010; Brandon-Mong et al. 2015). In recent years, various barcode libraries for numerous animal groups of Germany were established, including both marine and freshwater fish (Knebelsberger et al. 2014; Knebelsberger et al. 2015), amphibians and reptiles (Hawlitschek et al. 2016), echinoderms (Laakmann et al. 2017), molluscs (Gebhardt and Knebelsberger 2015; Barco et al. 2016), crustaceans (Raupach et al. 2015), spiders (Astrin et al. 2016), myriapods (Spelda et al. 2011), and numerous insect taxa, e.g., Coleoptera (Hendrich et al. 2015), Ephemeroptera, Plecoptera, Trichoptera (Morinière et al. 2017), Heteroptera (Raupach et al. 2014; Havemann et al. 2018), Hymenoptera (Schmidt et al. 2015; Schmidt et al. 2017; Schmid-Egger et al. 2019), Lepidoptera (Hausmann et al. 2011), Neuroptera (Morinière et al. 2014), and Orthoptera (Hawlitschek et al. 2017). Previous studies also laid the groundwork of a comprehensive DNA barcode library for the ground beetles (Coleoptera: Carabidae) of Germany (Raupach et al. 2010; Raupach et al. 2011; Hendrich et al. 2015; Raupach et al. 2016; Raupach et al. 2018; Raupach et al. 2019).

**Figure 1.**
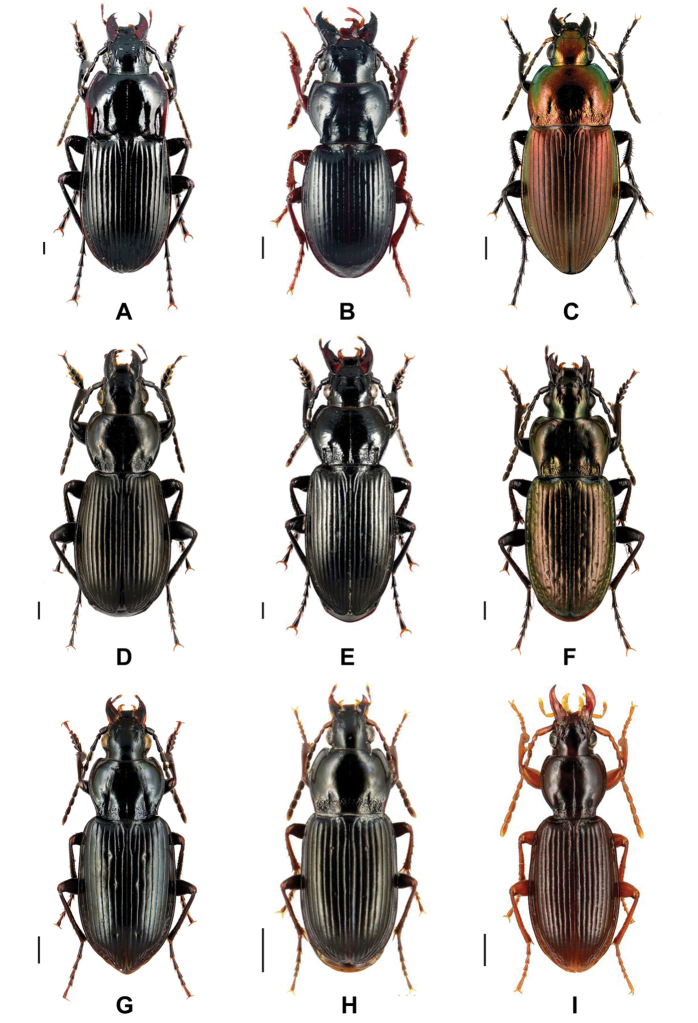
Representative images of analyzed beetle species **A***Abax parallelepipedus* (Piller & Mitterbacher, 1783) **B***Molops piceus* (Panzer, 1793) **C***Poecilus versicolor* (Sturm, 1824) **D**Pterostichus (Eosteropus) aethiops (Panzer, 1796) **E**Pterostichus (Omaseus) melanarius (Illiger, 1798) **F**Pterostichus (Oreophilus) multipunctatus (Dejean, 1828) **G**Pterostichus (Bothriopterus) quadrifoveolatus Letzner, 1852 **H**Pterostichus (Argutor) vernalis Panzer, 1796 and **I***Stomis pumicatus* (Panzer, 1795). Scale bars: 1 mm. All images were obtained from www.eurocarabidae.de.

The Carabidae are a cosmopolitan family with an estimated number of probably more than 40,000 species world-wide (Lindroth 1986). The margined pronotum, large head, prominent mandibles, and striate elytra help to characterize this family (Arnett and Thomas 2000). These features, however, vary considerably throughout this taxon. Ground beetles can be found in all habitats except deserts and polar regions. Most adults of this family present a somber appearance, that is, a uniformly dark color, although some species are bi- or tricolored dorsally, and can have striking patterns (e.g., *Callistus* Bonelli, 1810, *Omophron* Latreille, 1802 or *Panagaeus*, Latreille, 1804). Adult ground beetles range in size from 2 up to 70 mm (genus *Hyperion* Castelnau, 1834). Most carabids are predators of invertebrates and consume many pest species, and are therefore typically considered as beneficial organisms (e.g., Lövei 1996). Within the Carabidae, the genus *Pterostichus* Bonelli, 1810 is a very large and diverse taxon with a Holarctic distribution, also reaching the Oriental and Neotropical regions (Hůrka 1996). More than 1,000 species are known world-wide to date, with more than 200 species are recorded for Europe (Hůrka 1996; Luff 2007) and 36 documented in Germany, including some of the commonest carabids of Germany, e.g., *Pterostichus niger* (Schaller, 1783), *Pterostichus nigrita* (Paykull, 1790), or *Pterostichus strenuus* (Panzer, 1796) (Trautner et al. 2014). The genus is in the present concept, however, undoubtedly not monophyletic and has been subdivided into numerous subgenera or sometimes even genera in the past (e.g., Lindroth 1986; Hůrka 1996; Luff 2007). Unfortunately, a thorough and comprehensive revision is still missing. In order to accommodate this situation, the subgeneric arrangement used in this study follows the traditional arrangement (see Marggi 2006). In general, adults of the genus *Pterostichus* have a body length between 5 to 25 mm, with most species above average. They have normally a somewhat uniform appearance with a strongly sclerotized and stout pronotum, thick antennae, and rather long legs with pronounced tibiae (Lindroth 1986). The overwhelming majority of species are carnivorous, night-active black-colored insects; those with a metallic coloration are often diurnal (Lindroth 1986) (Fig. 1). Due to the fact that various species of this genus represent important and highly abundant elements of the carabid fauna of many habitats world-wide (e.g., Igondová and Majzlan 2015; Hong et al. 2017; Baranová et al. 2018), they have been intensively studied in the past, for example their general ecology (e.g., Rushton et al. 1990; Fournier and Loreau 2001; Allema et al. 2012; Brigić et al. 2014), feeding strategies (e.g., Symondson et al. 2000; Langan et al. 2001; Dinis et al. 2016), and zoogeography/phylogeography (e.g., Lagisz et al. 2010; Schmidt et al. 2012; Sasakawa et al. 2017).

In this study we present as part of the GBoL project a further step in generating a comprehensive DNA barcode library for the molecular identification of Central European ground beetle species, focusing on the genus *Pterostichus* and allied taxa. This barcode library included 36 species of *Pterostichus* as well as additional species of other genera belonging to the Pterostichini, including five species of the genus *Abax* Bonelli, 1810, three species of the genus *Molops* Bonelli, 1810, six species of the genus *Poecilus* Bonelli, 1810 and one species of the genus *Stomis* Clairville, 1806. In sum, 198 new barcodes were generated and a total number of 609 DNA barcodes examined in detail, including DNA barcodes of pinned museum specimens up to 39 years old.

## Materials and methods

### Sampling of specimens

Most studied ground beetles (n = 186, 93.9%) were collected between 2006 and 2018 using various sampling methods (i.e., hand collecting, pitfall traps). All beetles were stored in ethanol (96%). The analyzed specimens were identified using the identification key provided in Marggi (2006). It was also possible to generate DNA barcodes from pinned ground beetles of the carabid collection of the Bavarian State Collection of Zoology (n = 12, 6.1%), with an age between 22 and 39 years. In total, 198 new barcodes of 37 species were generated. Furthermore, we included 411 DNA barcodes from three previous studies (Raupach et al. 2010: 86 specimens, 16 species; Pentinsaari et al. 2014: 61 specimens, 15 species; Hendrich et al. 2015: 247 specimens, 37 species) and 17 sequences that were released without publication (7 species) in our analysis. Therefore, the complete dataset covered 609 DNA barcodes.

Most beetles were collected in Germany (n = 403, 66.2%), but for comparison various specimens were included from Austria (n = 82, 13.5%), Belgium (n = 16, 2.6%), Bulgaria (n = 1, 0.2%), Czech Republic (n = 4, 0.7%), Estonia (n = 2, 0.3%), Finland (n = 59, 9.7%), France (n = 16, 2.6%), Italy (n = 10, 1.6%), Romania (n = 2, 0.3%), Slovakia (n = 1, 0.2%), Slovenia (n = 10, 1.6%), and Switzerland (n = 3, 0.5%). The number of analyzed specimens per species ranged from one (6 species) to a maximum of 40 for *Poecilus versicolor* (Sturm, 1824).

### DNA barcode amplification, sequencing, and data depository

All laboratory operations were carried out, following standardized protocols for the cytochrome *c* oxidase subunit I (COI) fragment amplification and sequencing (Ivanova et al. 2006, deWaard et al. 2008), at the Canadian Center for DNA Barcoding (CCDB), University of Guelph, the molecular labs of the Zoologisches Forschungsmuseum Alexander Koenig in Bonn, the German Centre of Marine Biodiversity Research, Senckenberg am Meer, in Wilhelmshaven, and the working group Systematics and Evolutionary Biology at the Carl von Ossietzky University Oldenburg, all in Germany. Photos from each studied beetle were taken before molecular work started. One or two legs of one body side were removed for the subsequent DNA extraction which was performed using the QIAmp Tissue Kit (Qiagen GmbH, Hilden, Germany) or NucleoSpin Tissue Kit (Macherey-Nagel, Düren, Germany), following the manufacturer`s extraction protocol. The PCR temperature profile for the barcode fragment (approx. 660 base pairs) using the primer pair LCO1480 and HCO2198 (Folmer et al. 1994) consisted of an initial denaturation at 94 °C (5 min), followed by 38 cycles at 94 °C (denaturation, 45 s), 48 °C (annealing, 45 s), 72 °C (extension, 80 s), and a final extension 72 °C (7 min). Purified PCR products were cycle-sequenced and sequenced in both directions at contract sequencing facilities (Macrogen, Seoul, Korea, or GATC, Konstanz, Germany), using the same primers as used in PCR. Double stranded sequences were assembled and checked for mitochondrial pseudogenes (numts) analyzing the presence of stop codons, frameshifts as well as double peaks in chromatograms with Geneious Prime 2020.0.4 (https://www.geneious.com) (Biomatters, Auckland, New Zealand). For verification, BLAST searches (nBLAST, search set: others, program selection: megablast) were performed to confirm the identity of all new sequences as ground beetle barcodes based on already published sequences (high identity values, very low E-values).

Comprehensive voucher information, taxonomic classifications, photos, DNA barcode sequences, primer pairs used and trace files (including their quality) are publicly accessible through the public dataset “DS-BAPTE” (Dataset ID: dx.doi.org/10.5883/DS-BAPTE) on the Barcode of Life Data Systems (BOLD; www.boldsystems.org) (Ratnasingham and Hebert 2007). In addition, all new barcode data were deposited in GenBank (accession numbers: MN454529–MN454726).

### DNA barcode analysis

The complete dataset was analyzed by using an established workflow as it was already performed in former studies (Raupach et al. 2016, Raupach et al. 2018). The analysis tools of the BOLD workbench were employed to calculate the nucleotide composition of the sequences and distributions of Kimura-2-parameter distances (K2P; Kimura 1980) within and between species (align sequences: BOLD aligner; ambiguous base/gap handling: pairwise deletion). All barcode sequences became subject of the Barcode Index Number (BIN) analysis system implemented in BOLD that clusters DNA barcodes in order to produce operational taxonomic units that typically closely correspond to species (Ratnasingham and Hebert 2013). A first threshold of 2.2% was applied for a rough differentiation between intraspecific and interspecific distances, followed by refinements through Markov clustering into the final BINs (Ratnasingham and Hebert 2013). These BIN assignments on BOLD are constantly updated as new sequences are added, splitting and/or merging individual BINs in the light of new data (Ratnasingham and Hebert 2013).

In addition, all sequences were aligned using MUSCLE (Edgar 2004) and analyzed using a neighbor-joining cluster analysis (NJ; Saitou and Nei 1987) based on K2P distances with MEGA 10.0.5 (Kumar et al. 2018). Non-parametric bootstrap support values were obtained by resampling and analyzing 1,000 replicates (Felsenstein 1985). It should be explicitly noted that this analysis is not intended to be phylogenetic. Instead of this, the shown topology represents a graphical visualization of DNA barcode distance divergences and species clustering. For species pairs with interspecific distances < 2.2%, maximum parsimony networks were constructed with TCS 1.21 based on default settings (Clement et al. 2000), implemented in the software package PopART v.1.7 (Leigh and Bryant 2015). Such networks allow the identification of possible haplotype sharing between species as a consequence of recent speciation or on-going hybridization processes.

## Results

Overall, 609 DNA barcode sequences of 51 ground beetle species of the Pterostichini were analyzed. A full list of the analyzed species is presented in the supporting information (Suppl. material 1). For the genus *Pterostichus* we analyzed 31 species which represent 86% of all recorded species (n = 36) of this genus for Germany. Beside this, the given sampling covered four species of the genus *Abax* (recorded species for Germany: n = 4, therefore 100%), two species of the genus *Molops* (n = 2, 100%), six species of the genus *Poecilus* (n = 6, 100%), and the only known species for Germany of the genus *Stomis*. Seven additional analyzed species are actually not recorded from Germany but included for comparison: *Abax beckenhauptii* (Duftschmid, 1812) (n = 3), *Molops striolatus* (Fabricius, 1801) (n = 2), *Pterostichus adstrictus* Eschscholtz, 1823 (n = 5), *Pterostichus illigeri* (Panzer, 1803) (n = 3), *Pterostichus muehlfeldii* (Duftschmid, 1812) (n = 3), *Pterostichus schmidtii* (Chaudoir, 1838) (n = 3), and *Pterostichus ziegleri* (Duftschmid, 1812) (n = 8).

**Figure 2.**
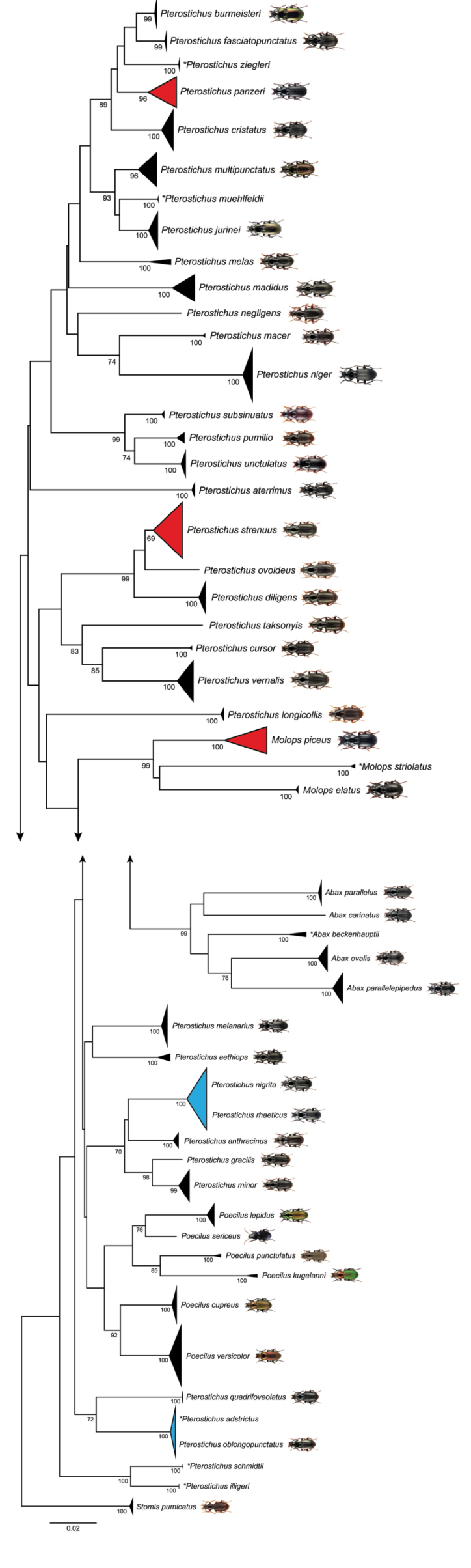
Neighbor-joining (NJ) topology of the analyzed ground beetle species based on Kimura 2-parameter distances. Triangles show the relative number of individual’s sampled (height) and sequence divergence (width). Red triangles indicate species with intraspecific maximum pairwise distances > 2.2%, blue triangles species pairs with interspecific distances < 2.2%. Numbers next to nodes represent non-parametric bootstrap values > 90% (1,000 replicates). Images are provided for species recorded in Germany whereas asterisks indicate species not recorded in Germany. All beetle images were obtained from www.eurocarabidae.de except of *Poecilus sericeus* (photographer: Katja Neven, Lars Hendrich).

In total, fragment lengths of the analyzed DNA barcode fragments ranged from 420 to 658 base pairs. As it is typically known for arthropods, the DNA barcode region was characterized by a high AT-content: the mean sequence compositions were A = 29.7%, C = 15.1%, G = 16.2%, and T = 39%. Intraspecific K2P distances ranged from 0 to a maximum of 3.15% (*Molops piceus*), whereas interspecific distances within the analyzed species had values between 0 and 11.19%. Lowest interspecific distances were found for *Pterostichus adstrictus* Eschscholtz, 1823 and *Pterostichus oblongopunctatus* (Fabricius, 1787) (0%; BIN: ABY4764) as well as *Pterostichus nigrita* Paykull, 1790 and *Pterostichus rhaeticus* Heer, 1837 (0%; BIN: AAM9738). In total, unique BINs were revealed for 44 species (86%), two BINs for two species (4%), three BINs for one species (2%) and one BIN for two species for two species pairs (8%). Due to the fact that the numbers of unspecified nucleotides (“Ns”) exceeds more than 1% of their total length, a distinct cluster of two sequences for *Pterostichus panzeri* (Panzer, 1803) received no BIN. The NJ analyses based on K2P distances revealed non-overlapping clusters with bootstrap support values > 95% for 40 species (78%) with more than one analyzed specimen (Fig. 2). A more detailed topology of all analyzed specimens is presented in the supporting information (Suppl. material 2).

**Figure 3.**
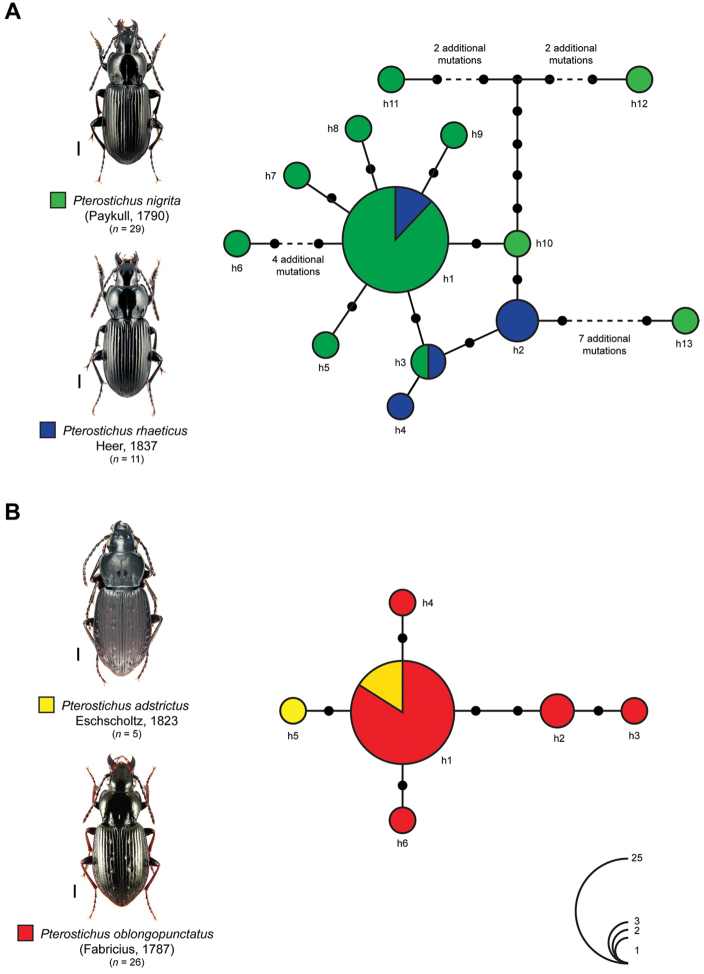
Maximum statistical parsimony networks of the sibling species pairs **A***Pterostichus nigrita* Paykull, 1790 (green) and *Pterostichus rhaeticus* Heer, 1837 (blue) and **B***Pterostichus adstrictus* Eschscholtz, 1823 (yellow) and *Pterostichus oblongopunctatus* (Fabricius, 1787) (red). Used parameters included default settings for connection steps, gaps were treated as fifth state. Each line represents a single mutational change whereas small black dots indicate missing haplotypes. The numbers of analyzed specimens (*n*) are listed, whereas the diameter of the circles is proportional to the number of haplotypes sampled (see given open circles with numbers). Scale bars 1 mm. Beetle images were obtained from www.eurocarabidae.de except *Pterostichus adstrictus* (photographer: Ditta Balke).

Our statistical maximum parsimony analysis showed multiple sharing of haplotypes for *Pterostichus nigrita* (n = 29)/*Pterostichus rhaeticus* (n = 11) and *Pterostichus adstrictus* (n = 5)/*Pterostichus oblongopunctatus* (n = 26) (Fig. 3). For *Pterostichus nigrita* and *Pterostichus rhaeticus* a number of 13 different haplotypes was found (Fig. 3A) One dominant haplotype (h1) was shared by 22 specimens of *Pterostichus nigrita* and three specimens of *Pterostichus rhaeticus* (Fig. 3A). Most other haplotypes, however, were revealed only for one specimen (singletons; *Pterostichus nigrita*: h5-h13, *Pterostichus rhaeticus*: h4) and located at the tips of the network, separated from haplotype h1 or other core haplotypes (h2, h3, h10) by up to nine additional mutational steps. In case of *Pterostichus adstrictus* and *Pterostichus oblongopunctatus*, the analysis identified six haplotypes (Fig. 3B). The dominant haplotype h1 was shared by four specimens of *Pterostichus adstrictus* and 21 specimens of *Pterostichus oblongopunctatus*, representing the 81% of the analyzed specimens. All others were connected to this haplotype by a maximum of four mutational steps in a star-like pattern, generating a compact network.

## Discussion

As a result of preservative, passionate and intensive work in the past centuries, carabid beetles have become one of the most prominent model groups of insects for biodiversity studies (Kotze et al. 2011). Generations of carabidologists clarified most aspects of their taxonomy and phylogeny, geographic distribution, habitat associations and ecological requirements, life history strategies and adaptations, in particular for those species found in Central Europe (Kotze et al. 2011). Due to the habitat specificity of various species, ground beetles are routinely used as biological indicators to assess land use changes among different ecosystems (Lövei and Sunderland 1996; Rainio and Niemelä 2003; Pearce and Venier 2004; Koivula 2011; Kotze et al. 2011).

The present study highlights the use of DNA barcodes for the identification of species of the five genera of Pterostichini found in Germany. Unique BINs were revealed for 44 species (86%) of the analyzed 51 taxa. This result coincides with high rates of successful species identification of previous barcoding studies in terms of carabid beetles (Raupach et al. 2010; Raupach et al. 2011; Pentinsaari et al. 2014; Hendrich et al. 2015; Raupach et al. 2018). Nevertheless, the data revealed some species pairs with low interspecific distances (< 2.2%) and shared haplotypes but also three species with intraspecific distances > 2.2%.

### Species with low interspecific variability

Interspecific distances with values below 2.2% were found for four ground beetle species pairs. Whereas *Pterostichus burmeisteri* Heer, 1838 and *Pterostichus fasciatopunctatus* (Creutzer, 1799) as well as *Pterostichus ovoideus* (Sturm, 1824) and *Pterostichus strenuus* (Panzer 1796) were characterized by distinct lineages, haplotype sharing was revealed for two species pairs that will be discussed more in detail in the following.

### The species complex *Pterostichus nigrita* Paykull, 1790/*Pterostichus rhaeticus* Heer, 1837

*Pterostichus rhaeticus* and *Pterostichus nigrita* of the subgenus Pseudomaseus Chaudoir, 1838 are commonly considered as closely related but distinct, sibling species (Koch and Thiele 1980; Koch 1984; Müller-Motzfeld and Hartmann 1985; Koch 1986; Angus et al. 2000). Both species have a Palearctic distribution and are found in Northern and Central Europe (e.g., Hůrka 1996; Marggi 2006; Trautner et al. 2014; Muilwijk et al. 2015), but have been also recorded on the Balkan recently (Brigić et al. 2014). They differ only in a few, subtle morphological features (Koch 1984, 1986; Angus et al. 2000, 2008; Brigić et al. 2014): Specimens of *Pterostichus rhaeticus* are typically smaller and narrower than those of *Pterostichus nigrita*, but in mixed populations, the differences in body size, length and width of the elytra were not observed and the overlap in sizes is considerable (Brigić et al. 2014). Furthermore, male specimens can be differentiated by the shape of the right paramere, which is larger for *Pterostichus nigrita* and is also characterized by a shallow incision (e.g., Hůrka 1996; Marggi 2006). In contrast to this, a deeper incision is found at the right paramere of *Pterostichus rhaeticus*. Nevertheless, considerable variations and intermediate forms have been documented in mixed populations of some regions and may limit the use of this character (e.g., Luff 1990; Angus et al. 2000; Brigić et al. 2014; Kendall 2017). In the case of females, both species can be distinguished by the form of the coxostylus and the shape of sclerotized part of the 8^th^ abdominal sternite (e.g., Koch 1986; Hůrka 1996; Angus et al. 2000). A previous study already showed that both species cannot be differentiated by the means of DNA barcodes based on shared haplotypes (Raupach et al. 2010). This result is supported by the analysis of additional data as part of this study (Fig. 3A). Various alternative hypothesis can explain these results. First, both species are distinct and do not hybridize, but lineage sorting has not been completed for the mitochondrial genome so far. Second, both species are not undergoing hybridization, but a relatively recent introgression event of the mitochondrial genome across the species boundary without concordant introgression of the nuclear genome took place. Third, extensive hybridization between both taxa is given, and these two forms might or might not be considered as different species. As consequence, additional fine scale ecological, morphological, morphometric as well as molecular data, in particular from the nuclear genome, have to be analyzed carefully to answer these questions in detail, with a focus on mixed populations from different localities.

### The species complex *Pterostichus*adstrictus Eschscholtz, 1823/*Pterostichus oblongopunctatus* (Fabricius, 1787)

All DNA barcodes data of *Pterostichus adstrictus* (n = 5) and some sequences of *Pterostichus oblongopunctatus* (n = 3) were part of a previous study but not discussed in detail (Pentinsaari et al. 2014). Both species belong to the subgenus Bothriopterus Chaudoir, 1835 and are considered as closely related but distinct species (Lindroth 1986; Persohn 1996; Luff 2007). Whereas *Pterostichus adstrictus* is an inhabitant of the Northern coniferous regions (e.g., of Sweden, Norway, North America, or North Britain), *Pterostichus oblongopunctatus* represents a common and widely distributed Euro-Siberian species that is typically found in eurytopic woodlands (Lindroth 1986). Both species are morphological highly similar and their ranges overlap broadly in Scandinavia, but specimens of *Pterostichus adstrictus* can be differentiated from those of *Pterostichus oblongopunctatus* by unicolored, usually black legs, and the wider pronotal side border (Lindroth 1986; Persohn 1996; Luff 2007). The already hypothesized close relationship of both species is supported by haplotype sharing of DNA barcodes (see h1) (Fig. 3B). Similar to the previously discussed species pair it is unclear if both species represent closely but distinct and “valid” species or hybridization events – which have not been documented so far – still take place.

### Species with high intraspecific variability

**Figure 4.**
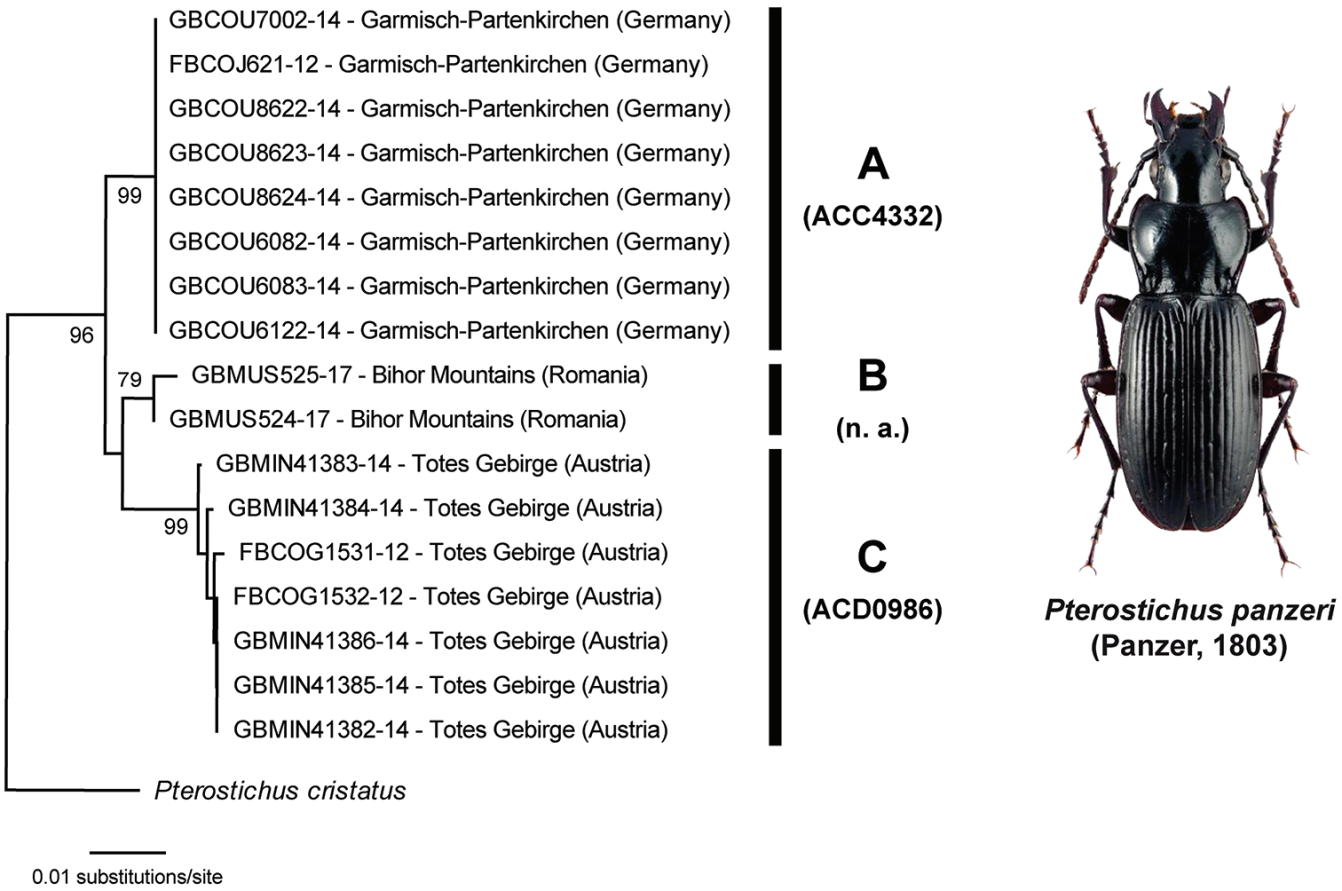
Subtree of the Neighbor-joining topology based on Kimura 2-parameter distances of all analyzed specimens of *Pterostichus panzeri* (Panzer, 1805) and nearest neighbor. Branches with specimen ID-number from BOLD, species names and sample localities. Numbers next to internal nodes are non-parametric bootstrap values (in %). Cluster (A-C) with BINs (if available) based on the barcode analysis from 11-05-2020. Beetle image was obtained from www.eurocarabidae.de.

**Figure 5.**
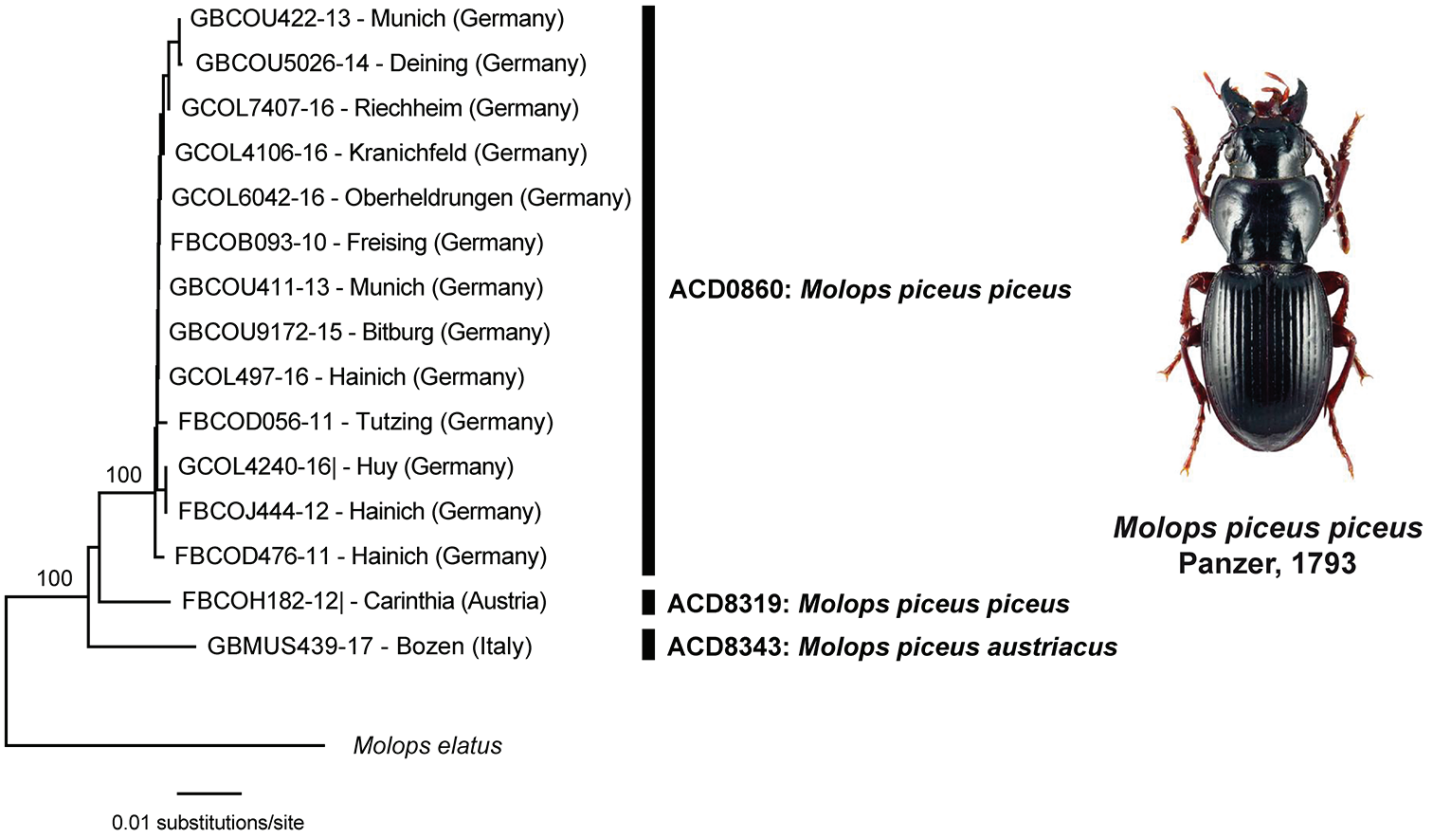
Subtree of the Neighbor-joining topology based on Kimura 2-parameter distances of all analyzed specimens of *Molops piceus* (Panzer, 1793) and nearest neighbor. Branches with specimen ID-number from BOLD, species names and sample localities. Numbers next to internal nodes are non-parametric bootstrap values (in %). Cluster (A-C) with BINs based on the barcode analysis from 11-05-2020. Beetle image was obtained from www.eurocarabidae.de.

Intraspecific pairwise distances with values > 2.2% were found for three species. Whereas *Pterostichus strenuus* (Panzer, 1796) showed no conspicuous substructure for the analyzed COI sequences (see Suppl. material 2), three clearly distinct monophyletic cluster/lineages were revealed within *Pterostichus panzeri* (Panzer, 1803) (Fig. 4, Table 1) and *Molops piceus* (Panzer, 1793) (Fig. 5, Table 1), respectively, and will be discussed more in detail.

**Table 1.** Intraspecific Kimura 2-distances for all distinct clusters of *Pterostichus panzeri* (Panzer, 1805).

	Cluster A (Germany) BIN: ACC4332	Cluster B (Romania) BIN: n. a.	Cluster C (Austria) BIN: ACD0986
**Cluster A (Germany) BIN: ACC4332**	0		
**Cluster B (Romania) BIN: n. a.**	0.014	0	
**Cluster C (Austria) BIN: ACD0986**	0.019 – 0.023	0.018 – 0.02	0 – 0.005

### Ménage à trois: three distinct clusters within *Pterostichus panzeri* (Panzer, 1803)

The carabid *Pterostichus panzeri* is a subalpine/alpine brachypterous species associated with chalk and distributed in the Central European mountain regions (e.g., Horion 1941; Holdhaus 1954; Müller-Kroehling 2013; Trautner and Rietze 2017). Interestingly, all three clusters correlate with different geographic localities: cluster A included all specimens (n = 8) from Garmisch-Partenkirchen (Germany) (BIN: ACC4332), cluster B contained only specimens from Bihor Mountains (Romania, n = 2, no BIN), and cluster C included beetles sampled in Austria (Totes Gebirge, n = 7, BIN: ACD0986) (Fig. 4). K2P distances between all clusters ranged from 1.4 to 2.3% (Table 1). Due to the fact that *Pterostichus panzeri* is associated to a specific habitat, it is likely that the observed genetic variability represents a result of phylogeographic effects. As a consequence of recurrent glaciation events, populations could have become isolated and gene flow disrupted, resulting in specific local haplotypes. Similar results have been found for other ground beetle species in the past (e.g., Zhang et al. 2006; Homburg et al. 2013; Faille et al. 2015). The existence of cryptic species, however, cannot be fully excluded, but no morphological variations between different populations have been reported so far. Furthermore, it should be also noted that the loss of the ability to fly can lead to a relaxed purifying selection on genes that are involved in the oxygen metabolism including COI, leading to accelerated rates of divergence in the barcode region within insects (Mitterboeck and Adamowicz 2013). The molecular analysis of additional specimens from other regions, as well as linkage groups in the nuclear genome, combined with thoroughly morphological studies will help to interpret the given results more in detail.

### *Molops piceus austriacus* Ganglbauer, 1889: not a subspecies but “real” species?

For *Molops piceus*, an oligophagous, brachypterous species that is found in forests, two subspecies are known: *Molops piceus piceus* Panzer 1793 and *Molops piceus austriacus* Ganglbauer, 1889. Whereas most analyzed beetles were specimens of the subspecies *Molops piceus piceus* (n = 14), only one specimen of *Molops piceus austriacus* was studied. Nevertheless, this beetle was clearly separated from all other specimens with high K2P distance values (BIN: ADO8343) (Fig. 5, Table 2). In the past, *Molops piceus austriacus* has been already considered as species (Marcuzzi 1956), but only the analysis of additional specimens, additional molecular markers (e.g., hypervariable elements of the nuclear rRNA genes (Raupach et al. 2010)) and careful morphological studies will help to clarify this taxonomic problem. Furthermore, all beetles of *Molops piceus piceus* from Germany (n = 12) (BIN: ADO0860) were separated from one animal collected in Carinthia (Austria) (BIN: ADO8319), highlighting the necessity of additional comprehensive morphological and molecular analysis for this species.

**Table 2.** Intraspecific Kimura 2-distances for all distinct clusters of *Molops piceus* (Panzer, 1793).

	***M. piceus piceus* (Germany) BIN: ADO0860**	***M. piceus piceus* (Austria) BIN: ADO8319**	***M. piceus austriacus* (Italy) BIN: ADO8343**
***M. piceus piceus* (Germany) BIN: ADO0860**	0–0.003		
***M. piceus piceus* (Austria) BIN: ADO8319**	0.019–0.028	0	
***M. piceus austriacus* (Italy) BIN: ADO8343**	0.029–0.032	0.028	0

## Conclusions

The build-up of comprehensive DNA barcode libraries represents a pivotal task for modern molecular biodiversity research and species surveys (e.g., Brandon-Mong et al. 2015, Curry et al. 2018). This is especially true for the hyperdiverse and numerous species of insects. Within the beetles, carabids are highly valuable bioindicators that are used routinely to characterize disturbances in various habitats such as forests, meadows, river banks, or fens for a long time. Our DNA barcode library clearly encourages the application of DNA barcodes as effective method for the molecular identification of species of *Pterostichus* and allied taxa even if a few species pairs cannot be resolved. The given data, however, also revealed distinct lineages that correlate with high distances within a few species, indicating significant phylogeographic patterns and/or even the possible existence of overlooked cryptic species.

## Supplementary materials

Supplementary material 1 Barcode analysis using the BOLD workbench Data type Data table Explanation note Molecular distances based on the Kimura 2-parameter model of the analyzed specimens of the analyzed species of the genera *Abax*, *Molops*, *Poecilus*, *Pterostichus*, and *Stomis*. Divergence values were calculated for all studied sequences, using the Nearest Neighbour Summary implemented in the Barcode Gap Analysis tool provided by the Barcode of Life Data System (BOLD). Align sequencing option: BOLD aligner (amino acid based HMM), ambiguous base/gap handling: pairwise deletion. ISD = intraspecific distance. BINs are based on the barcode analysis from 11-05-2020. Asterisks indicate species not recorded from Germany. Species with intraspecific maximum pairwise distances > 2.2% and species pairs with interspecific distances < 2.2% are marked in bold. As consequence that the numbers of unspecified nucleotides (“Ns”) exceeds more than 1% of their total length, a distinct cluster with two sequences for *Pterostichus panzeri* (Panzer, 1803) received no BIN. Country codes (alpha-2 code): AT = Austria, BE = Belgium, BG = Bulgaria, CZ = Czech Republic, EE = Estonia, FI = Finland, FR = France, DE = Germany, IT = Italy, RO = Romania, SK = Slovakia, SL = Slovenia and CH = Switzerland. https://binary.pensoft.net/file/468263This dataset is made available under the Open Database License (http://opendatacommons.org/licenses/odbl/1.0/). The Open Database License (ODbL) is a license agreement intended to allow users to freely share, modify, and use this Dataset while maintaining this same freedom for others, provided that the original source and author(s) are credited.Michael J. Raupach, Karsten Hannig, Jérome Morinière, Lars Hendrich

Supplementary material 2 Neighbor-joining topology Data type Neighbor-joining topology Explanation note Neighbor-joining topology of all analyzed carabid beetles based on Kimura 2-parameter distances. Specimens are classified using ID numbers from BOLD and species name. Numbers next to nodes represent non-parametric bootstrap values (1,000 replicates, in %). *Pterostichus crenatus* (Duftschmid, 1812) (see five specimens from Pentinsaari et al 2014) is a junior synonym of *Pterostichus vernalis* (Panzer, 1795). Furthermore, a specimen of *Pterostichus hagenbachii* (Sturm, 1824) (see Hendrich et al. 2015) was incorrectly determined. A careful re-inspection revealed this specimen as *Pterostichus cristatus* (Dufour, 1820). https://binary.pensoft.net/file/468264This dataset is made available under the Open Database License (http://opendatacommons.org/licenses/odbl/1.0/). The Open Database License (ODbL) is a license agreement intended to allow users to freely share, modify, and use this Dataset while maintaining this same freedom for others, provided that the original source and author(s) are credited.Michael J. Raupach, Karsten Hannig, Jérome Morinière, Lars Hendrich
