# Supplementary material for: A DNA barcode library for ground beetles of Germany: the genus Pterostichus Bonelli, 1810 and allied taxa (Insecta, Coleoptera, Carabidae)
Source: Zookeys. 2020 Oct 28;980:93–117. doi: 10.3897/zookeys.980.55979 (PMC7642132; doi:10.3897/zookeys.980.55979)
Supplement: Supplementary material 1 — Barcode analysis using the BOLD workbench [file zookeys-980-093-s001.docx]

| No. | Species | Mean ISD | Max ISD | *n* | BIN | Country^1^ | Nearest Species | Distance to NN |
| --- | --- | --- | --- | --- | --- | --- | --- | --- |
| 1 | **Abax beckenhaupti* (Duftschmid, 1812) | 1.03 | 1.55 | 3 | ACM7168 | AT | *Abax parallelepipedus* | 9.14 |
|  | *Abax carinatus* (Duftschmid, 1812) | 0 | 0 | 1 | ACD1123 | SL | *Abax paralleus* | 10.34 |
|  | *Abax ovalis* (Duftschmid, 1812) | 0.25 | 1.39 | 17 | AAN9386 | BG, DE, SL | *Abax parallelepipedus* | 8.41 |
|  | *Abax parallelepipedus* (Piller & Mitterpacher, 1783) | 0.17 | 1.09 | 20 | AAN9385 | BG, FR, DE, SL | *Abax ovalis* | 8.41 |
| 5 | *Abax parallelus* (Duftschmid, 1812) | 0.16 | 0.46 | 16 | AAO0357 | AT, DE | *Abax beckenhauptii* | 9.99 |
|  |  |  |  |  |  |  |  |  |
| 1 | *Molops elatus* Fabricius, 1801 | 0.08 | 0.15 | 4 | ACB8956 | DE | *Molops piceus* | 8.66 |
|  | ***Molops piceus* Panzer, 1793** | **0.73** | **3.15** | **15** | **ADO8319, ADO8343, AAO0860** | **AT, DE, IT** | ***Molops elatus*** | **8.66** |
|  | **Molops striolatus* (Fabricius, 1801) | 0.31 | 0.31 | 2 | ACR8677 | SL | *Molops piceus* | 11.19 |
|  |  |  |  |  |  |  |  |  |
| 1 | *Poecilus cupreus* (Linnaeus, 1758) | 0.31 | 1.26 | 24 | AAF0283 | DE, FI | *Poecilus versicolor* | 4.6 |
|  | *Poecilus kugelanni* (Panzer, 1797) | 0.27 | 0.27 | 2 | ADP1132 | FR | *Poecilus punctulatus* | 4.98 |
|  | *Poecilus lepidus* (Leske, 1785) | 0.28 | 0.69 | 15 | AAY7564 | DE, FR, IT | *Poecilus sericeus* | 3.9 |
|  | *Poecilus punctulatus* (Schaller, 1783) | 0.22 | 0.22 | 2 | ADP3430 | AT | *Poecilus lepidus* | 4.69 |
| 5 | *Poecilus sericeus* Fischer von Waldheim, 1824 | 0 | 0 | 1 | ADP2701 | AT | *Poecilus lepidus* | 3.9 |
|  | *Poecilus versicolor* (Sturm, 1824) | 0.35 | 1.17 | 40 | AAH9293 | AT, DE, EE, FR, FI, SL | *Poecilus cupreus* | 4.6 |
|  |  |  |  |  |  |  |  |  |
| 1 | ****Pterostichus (Bothriopterus) adstrictus* Eschscholtz, 1823** | **0.06** | **0.15** | **5** | **ABY4764** | **FI** | ***Pterostichus oblongopunctatus*** | **0** |
|  | *Pterostichus (Eosteropus) aethiops* (Panzer, 1796) | 0.52 | 1.32 | 5 | AAY8411 | DE | *Pterostichus strenuus* | 5.83 |
|  | *Pterostichus (Pseudomaseus) anthracinus* (Illiger, 1798) | 0.09 | 0.46 | 10 | AAO1075 | DE | *Pterostichus gracilis* | 4.25 |
|  | *Pterostichus (Melanius) aterrimus* (Herbst, 1784) | 0.27 | 1.08 | 8 | AAY7522 | BE, DE | *Pterostichus anthracinus* | 8.58 |
| 5 | ***Pterostichus (Cheropus) burmeisteri* Heer, 1838** | **0.04** | **0.32** | **17** | **AAX7561** | **AT, DE** | ***Pterostichus fasciatopunctatus*** | **1.61** |
|  | *Pterostichus (Pterostichus) cristatus* (Dufour, 1820)^2^ | 0.36 | 1.24 | 23 | ACG2758 | BE, DE, FR, IT, CH | *Pterostichus burmeisteri* | 3.15 |
|  | *Pterostichus (Argutor) cursor* (Dejean, 1828) | 0.15 | 0.15 | 2 | ADP2300 | AT | *Pterostichus vernalis* | 6.03 |
|  | *Pterostichus (Phonias) diligens* (Sturm, 1824) | 0.31 | 0.82 | 18 | ACG2755 | DE, FI | *Pterostichus ovoideus* | 3.84 |
|  | ***Pterostichus (Pterostichus) fasciatopunctatus* (Creutzer, 1799)** | **0.05** | **0.31** | **12** | **ABY6718** | **AT, DE, SL** | ***Pterostichus burmeisteri*** | **1.61** |
| 10 | *Pterostichus (Pseudomaseus) gracilis* (Dejean, 1828) | 0 | 0 | 1 | AAY8416 | DE | *Pterostichus minor* | 2.47 |
|  | **Pterostichus (Pseudosteropus) illigeri* (Panzer, 1803) | 0 | 0 | 3 | ACM7956 | AT | *Pterostichus schmidtii* | 4.37 |
|  | *Pterostichus (Oreophilus) jurinei* (Panzer, 1803) | 0.17 | 0.77 | 21 | ABA3646 | AT, IT | *Pterostichus multipunctatus* | 2.31 |
|  | *Pterostichus (Pedius) longicollis* (Duftschmid, 1812) | 0.12 | 0.31 | 7 | ACC4432 | CZ, DE, FR | *Poecilus sericeus* | 10.05 |
|  | *Pterostichus (Adelosia) macer* (Marsham, 1802) | 0.15 | 0.15 | 2 | ACC9854 | DE | *Pterostichus multipunctatus* | 7.14 |
| 15 | *Pterostichus (Pterostichus) madidus* (Fabricius, 1775) | 0.51 | 1.45 | 15 | ACT9224, AAH0299 | BE, DE, FR | *Pterostichus multipunctatus* | 5.77 |
|  | *Pterostichus (Morphnosoma) melanarius* (Illiger, 1798) | 0.36 | 1.55 | 27 | AAC0661 | EE, FR, FI, DE | *Pterostichus aethiops* | 6.09 |
|  | *Pterostichus (Feronidius) melas* (Creutzer, 1799) | 1.14 | 1.71 | 3 | AAX7565 | DE, SK, SL | *Pterostichus fasciatopunctatus* | 4.93 |
|  | *Pterostichus (Pseudomaseus) minor* (Gyllenhal, 1827) | 0.34 | 1 | 21 | AAX7566 | BE, DE, FI | *Pterostichus gracilis* | 2.47 |
|  | **Pterostichus (Cheropus) muehlfeldii* (Duftschmid, 1812) | 0 | 0 | 3 | ACT8282 | AT | *Pterostichus multipunctatus* | 2.37 |
| 20 | *Pterostichus (Oreophilus) multipunctatus* (Dejean, 1828) | 0.36 | 1.27 | 19 | AAZ5408 | DE, FR, CH | *Pterostichus jurinei* | 2.31 |
|  | *Pterostichus (Cryobius) negligens* (Sturm, 1824) | 0 | 0 | 1 | ADO9172 | BG | *Pterostichus fasciatopunctatus* | 6.22 |
|  | *Pterostichus (Platysma) niger* (Schaller, 1783) | 0.2 | 0.93 | 30 | AAL2225 | BE, DE, FI | *Pterostichus macer* | 7.5 |
|  | ***Pterostichus (Pseudomaseus) nigrita* (Paykull, 1790)** | **0.27** | **1.93** | **29** | **AAM9738** | **DE, FI, SL** | ***Pterostichus rhaeticus*** | **0** |
|  | ***Pterostichus (Bothriopterus) oblongopunctatus* (Fabricius, 1787)** | **0.1** | **0.62** | **26** | **ABY4764** | **BE, DE, FI** | ***Pterostichus adstrictus*** | **0** |
| 25 | ***Pterostichus (Phonias) ovoideus* (Sturm, 1824)** | **0** | **0** | **1** | **AAX7609** | **DE** | ***Pterostichus strenuus*** | **1.97** |
|  | ***Pterostichus (Platypterus) panzeri* (Panzer, 1803)** | **1.17** | **2.34** | **17** | **ACC4332, ACD0986^4^** | **AT, DE** | ***Pterostichus burmeisteri*** | **2.79** |
|  | *Pterostichus (Cryobius) pumilio* (Dejean, 1828) | 0.3 | 0.65 | 6 | ABX2992 | DE | *Pterostichus unctulatus* | 3.12 |
|  | *Pterostichus (Bothriopterus) quadrifoveolatus* Letzner, 1852 | 0.04 | 0.16 | 8 | ACC3128 | DE | *Pterostichus oblongopunctatus* | 7.04 |
|  | ***Pterostichus (Pseudomaseus) rhaeticus* Heer, 1837** | **0.69** | **1.87** | **11** | **AAM9738** | **DE, FI** | ***Pterostichus nigrita*** | **0** |
| 30 | **Pterostichus (Pseudosteropus) schmidtii* (Chaudoir, 1838) | 0 | 0 | 3 | ACR2377 | AT | *Pterostichus illigeri* | 4.37 |
|  | ***Pterostichus (Phonias) strenuus* (Panzer, 1796)** | **0.53** | **2.21** | **31** | **AAO1071** | **BE, DE, FI** | ***Pterostichus ovoideus*** | **1.97** |
|  | *Pterostichus (Cryobius) subsinuatus* (Dejean, 1828) | 0.08 | 0.15 | 4 | ACD0583 | AT | *Pterostichus pumilio* | 3.12 |
|  | *Pterostichus (Phonias) taksonyis* Csiki, 1930 | 0 | 0 | 1 | ADP0501 | AT | *Pterostichus vernalis* | 7.58 |
|  | *Pterostichus (Cryobius) unctulatus* (Duftschmid, 1812) | 0.1 | 0.32 | 15 | AAZ9100 | AT, DE, IT | *Pterostichus pumilio* | 3.12 |
| 35 | *Pterostichus (Argutor) vernalis* (Panzer, 1796)^3^ | 0.12 | 1.41 | 23 | AAM0036 | DE, FI | *Pterostichus cursor* | 6.03 |
|  | **Pterostichus (Platypterinus) ziegleri* (Duftschmid, 1812) | 0.04 | 0.15 | 8 | AAM0035 | AT | *Pterostichus burmeisteri* | 2.79 |
|  |  |  |  |  |  |  |  |  |
| 1 | *Stomis pumicatus* (Panzer, 1795) | 0.11 | 0.31 | 11 | AAL9921 | DE, FI | *Poecilus punctualtus* | 10.66 |

**Notes**

1: Country codes (alpha-2 code): AT = Austria, BE = Belgium, BG = Bulgaria, CZ = Czech Republic, EE = Estonia, FI = Finland, FR = France, DE = Germany, IT = Italy, RO = Romania, SK = Slovakia, SL = Slovenia and CH = Switzerland.

2: A beetle of a previous publication that was identified as *Pterostichus hagenbachii* (Sturm, 1824) (Hendrich et al. 2015) was incorrectly determined. A careful inspection revealed this specimen as *Pterostichus cristatus* (Dufour, 1820).

3: *Pterostichus crenatus* (Duftschmid, 1812) (see five specimens from Pentinsaari et al 2014) is a junior synonym of *Pterostichus vernalis* (Panzer, 1795).

4: Due to the fact that the numbers of unspecified nucleotides (“Ns”) exceeds more than 1% of their total length, a distinct cluster that consists of old museum specimens with two sequences received no BIN.

**References**

**Hendrich L, Morinière J, Haszprunar G, Hebert PDN, Hausmann A, Köhler F, Balke M (2015) A comprehensive DNA barcode database for Central European beetles with a focus on Germany: Adding more than 3,500 identified species to BOLD. Molecular Ecology Resources 15: 795-818.** doi:10.1111/1755-0998

Marggi W (2006) Bd. 2, Adephaga 1: Carabidae (Laufkäfer). In: Freude H, Harde KW, Lohse GA, Klausnitzer B (Eds) Die Käfer Mitteleuropas. Spektrum-Verlag, Heidelberg/Berlin, 521 pp. In German.

Pentinsaari M, Hebert PDN, Mutanen M (2014) Barcoding beetles: a regional survey of 1872 species reveals high identification success and unusually deep interspecific divergences. Public Library of Science ONE 9: e108651. doi:10.1371/journal.pone.0108651
