# Supplementary material for: A DNA barcode library for ground beetles of Germany: the genus Pterostichus Bonelli, 1810 and allied taxa (Insecta, Coleoptera, Carabidae)
Source: Zookeys. 2020 Oct 28;980:93–117. doi: 10.3897/zookeys.980.55979 (PMC7642132; doi:10.3897/zookeys.980.55979)
Supplement: Supplementary material 2 — Neighbor-joining topology [file zookeys-980-093-s002.pdf]

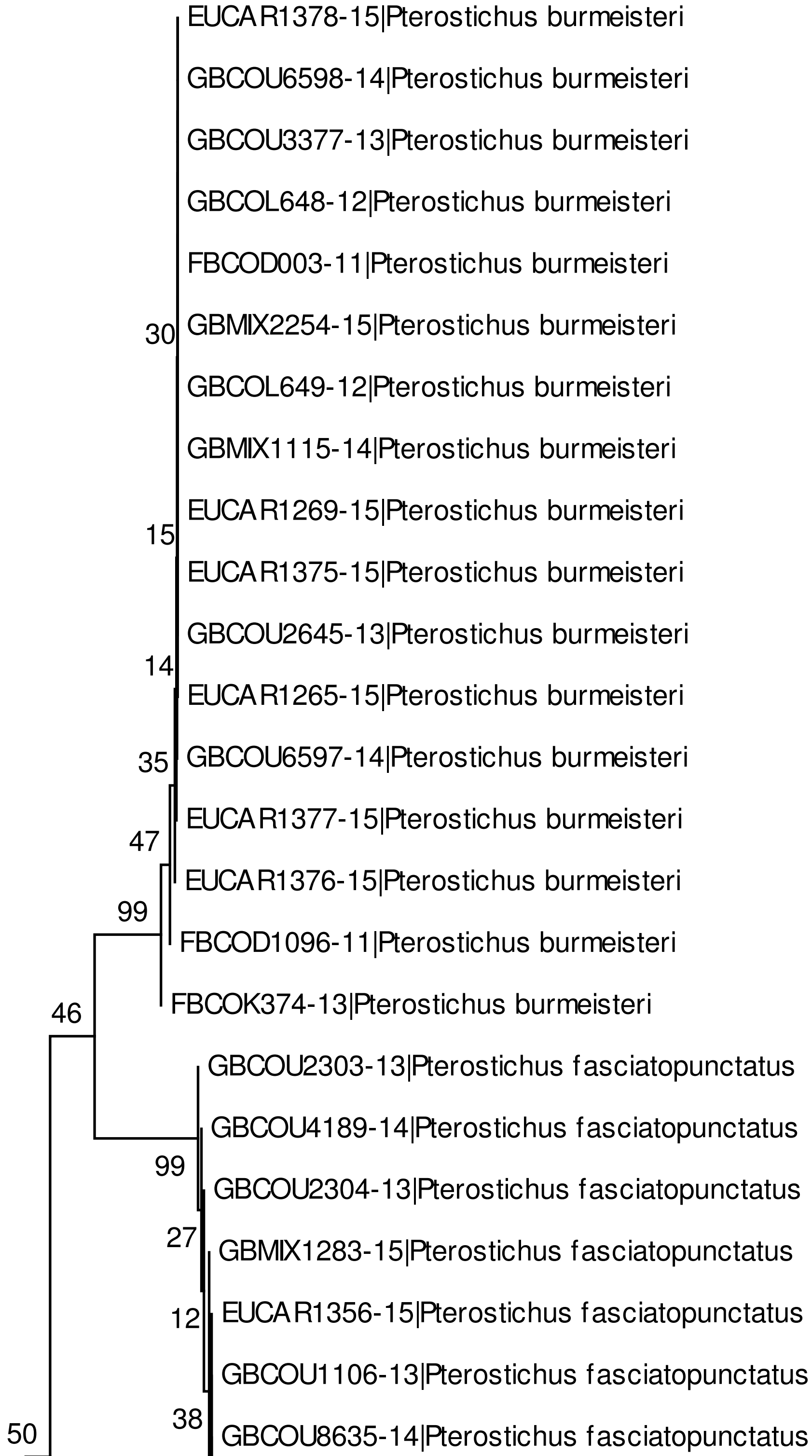
EUCAR1378-15|Pterostichus burmeisteri
GBCOU6598-14|Pterostichus burmeisteri
GBCOU3377-13|Pterostichus burmeisteri
GBCOL648-12|Pterostichus burmeisteri
FBCOD003-11|Pterostichus burmeisteri
GBMIX2254-15|Pterostichus burmeisteri
30
GBCOL649-12|Pterostichus burmeisteri
GBMIX1115-14|Pterostichus burmeisteri
EUCAR1269-15|Pterostichus burmeisteri
15
EUCAR1375-15|Pterostichus burmeisteri
GBCOU2645-13|Pterostichus burmeisteri
14
EUCAR1265-15|Pterostichus burmeisteri
35
GBCOU6597-14|Pterostichus burmeisteri
EUCAR1377-15|Pterostichus burmeisteri
47
EUCAR1376-15|Pterostichus burmeisteri
99
FBCOD1096-11|Pterostichus burmeisteri
FBCOK374-13|Pterostichus burmeisteri
46
GBCOU2303-13|Pterostichus fasciatopunctatus
GBCOU4189-14|Pterostichus fasciatopunctatus
99
GBCOU2304-13|Pterostichus fasciatopunctatus
27
GBMIX1283-15|Pterostichus fasciatopunctatus
12
EUCAR1356-15|Pterostichus fasciatopunctatus
GBCOU1106-13|Pterostichus fasciatopunctatus
38
GBCOU8635-14|Pterostichus fasciatopunctatus
50

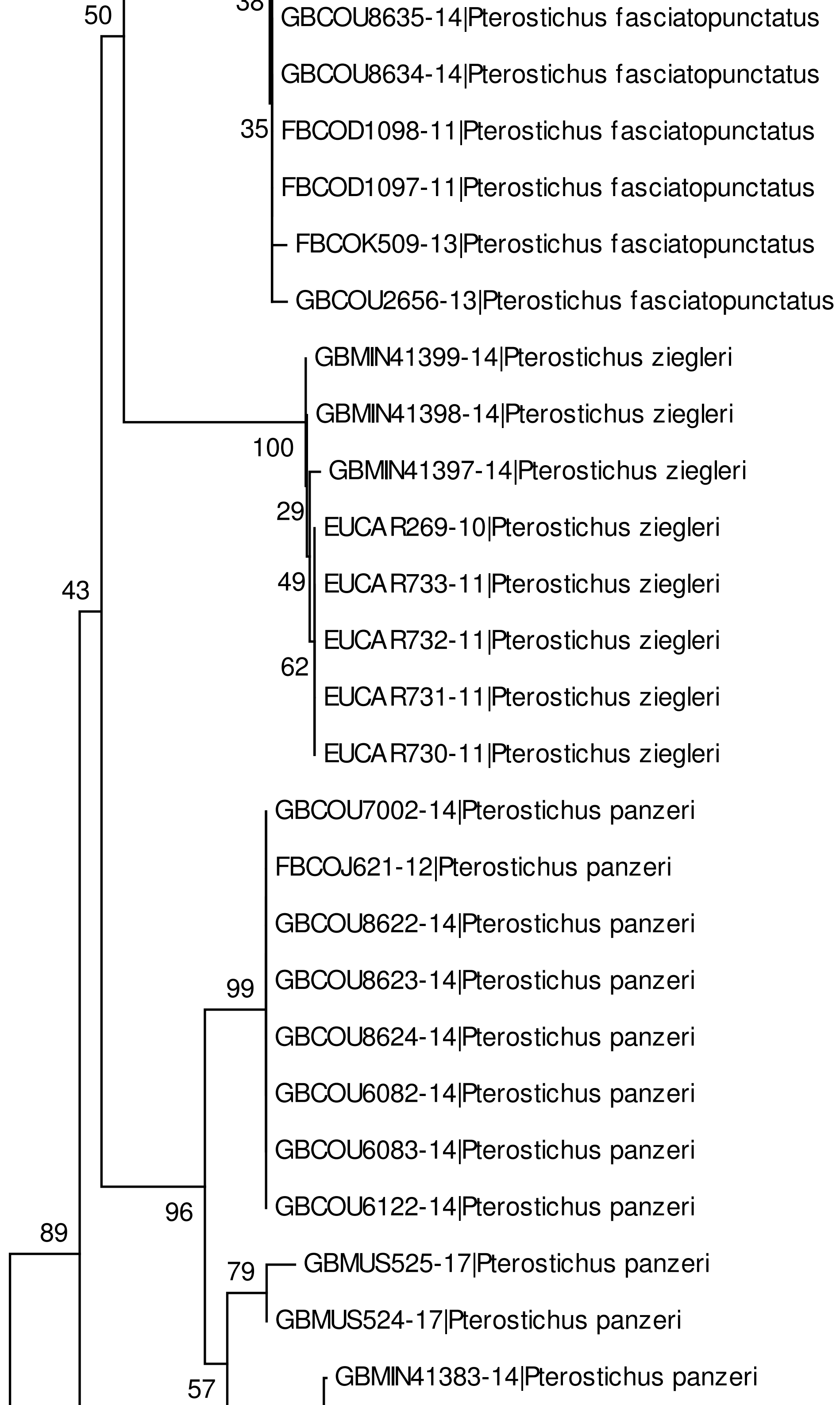
50
38
GBCOU8635-14|Pterostichus fasciatopunctatus
GBCOU8634-14|Pterostichus fasciatopunctatus
35
FBCOD1098-11|Pterostichus fasciatopunctatus
FBCOD1097-11|Pterostichus fasciatopunctatus
FBCOK509-13|Pterostichus fasciatopunctatus
GBCOU2656-13|Pterostichus fasciatopunctatus
GBMIN41399-14|Pterostichus ziegleri
GBMIN41398-14|Pterostichus ziegleri
100
GBMIN41397-14|Pterostichus ziegleri
29
EUCAR269-10|Pterostichus ziegleri
43
49
EUCAR733-11|Pterostichus ziegleri
EUCAR732-11|Pterostichus ziegleri
62
EUCAR731-11|Pterostichus ziegleri
EUCAR730-11|Pterostichus ziegleri
GBCOU7002-14|Pterostichus panzeri
FBCOJ621-12|Pterostichus panzeri
GBCOU8622-14|Pterostichus panzeri
99
GBCOU8623-14|Pterostichus panzeri
GBCOU8624-14|Pterostichus panzeri
GBCOU6082-14|Pterostichus panzeri
GBCOU6083-14|Pterostichus panzeri
96
GBCOU6122-14|Pterostichus panzeri
89
79
GBMUS525-17|Pterostichus panzeri
GBMUS524-17|Pterostichus panzeri
57
GBMIN41383-14|Pterostichus panzeri

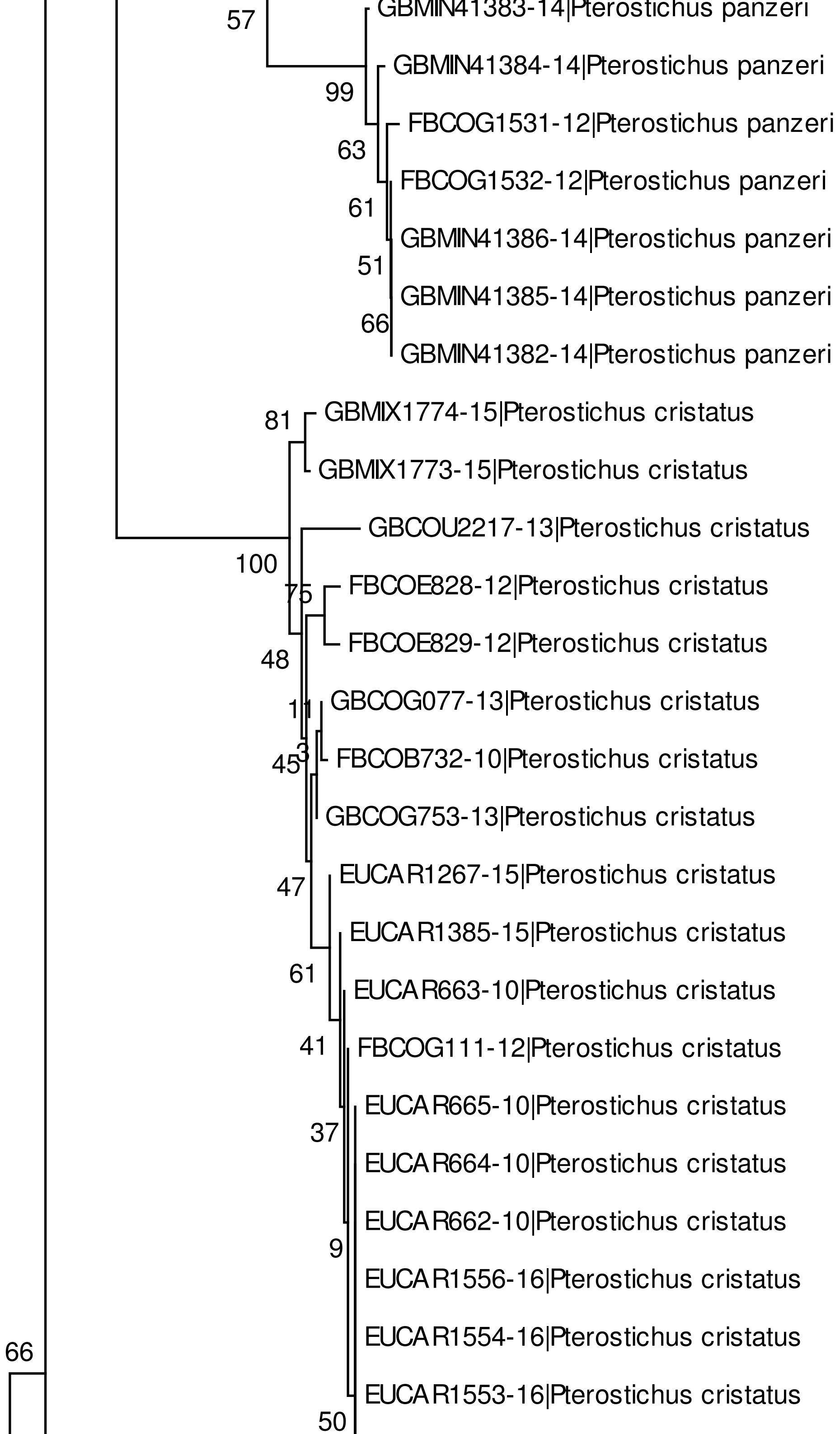
GBMIN41383-14|Pterostichus panzeri
GBMIN41384-14|Pterostichus panzeri
FBCOG1531-12|Pterostichus panzeri
FBCOG1532-12|Pterostichus panzeri
GBMIN41386-14|Pterostichus panzeri
GBMIN41385-14|Pterostichus panzeri
GBMIN41382-14|Pterostichus panzeri
GBMIX1774-15|Pterostichus cristatus
GBMIX1773-15|Pterostichus cristatus
GBCOU2217-13|Pterostichus cristatus
FBCOE828-12|Pterostichus cristatus
FBCOE829-12|Pterostichus cristatus
GBCOG077-13|Pterostichus cristatus
FBCOB732-10|Pterostichus cristatus
GBCOG753-13|Pterostichus cristatus
EUCAR1267-15|Pterostichus cristatus
EUCAR1385-15|Pterostichus cristatus
EUCAR663-10|Pterostichus cristatus
FBCOG111-12|Pterostichus cristatus
EUCAR665-10|Pterostichus cristatus
EUCAR664-10|Pterostichus cristatus
EUCAR662-10|Pterostichus cristatus
EUCAR1556-16|Pterostichus cristatus
EUCAR1554-16|Pterostichus cristatus
EUCAR1553-16|Pterostichus cristatus
57
99
63
61
51
66
81
100
75
48
11
3
45
47
61
41
37
9
66
50

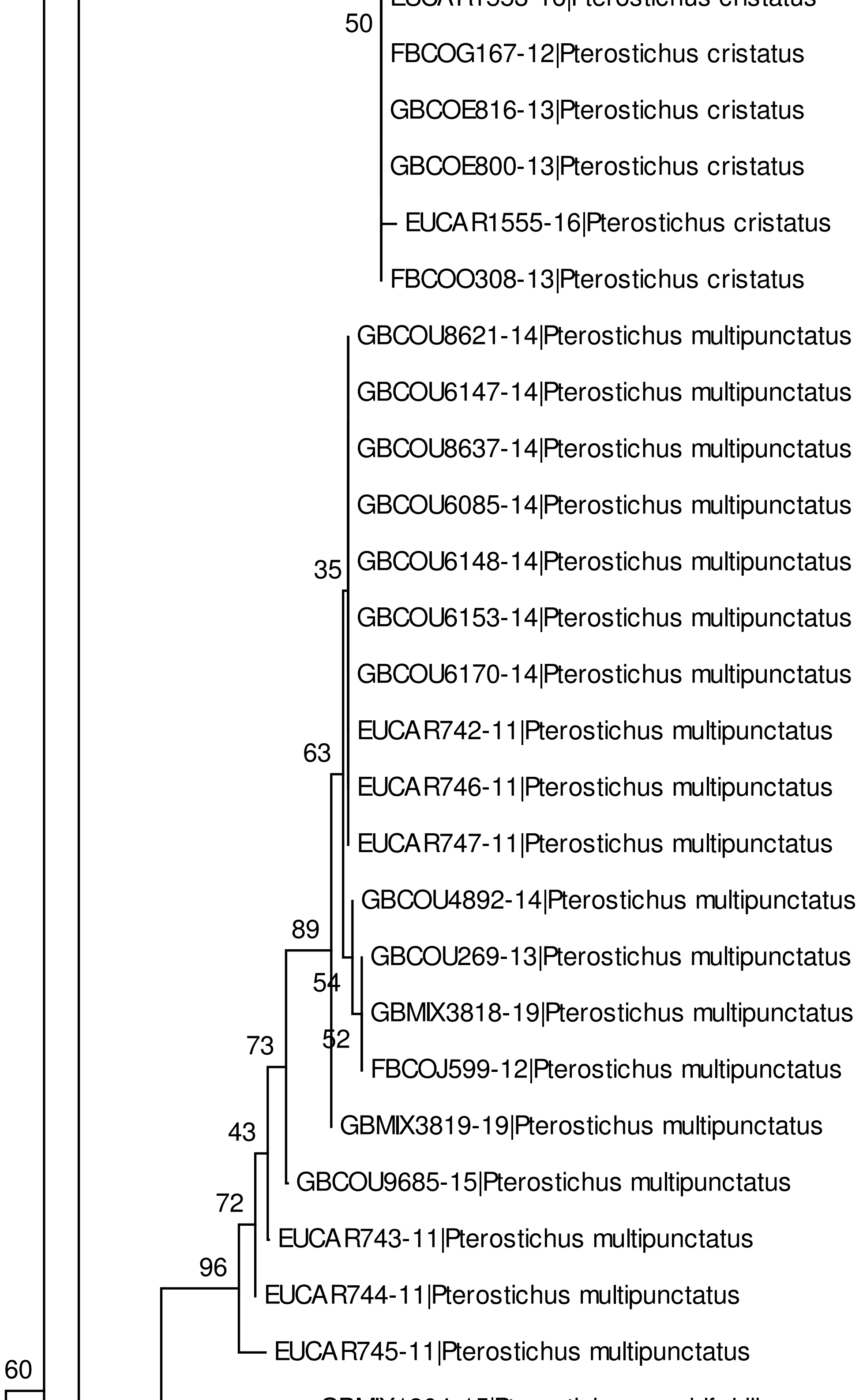

50
FBCOG167-12|Pterostichus cristatus
GBCOE816-13|Pterostichus cristatus
GBCOE800-13|Pterostichus cristatus
EUCAR1555-16|Pterostichus cristatus
FBCOO308-13|Pterostichus cristatus
GBCOU8621-14|Pterostichus multipunctatus
GBCOU6147-14|Pterostichus multipunctatus
GBCOU8637-14|Pterostichus multipunctatus
GBCOU6085-14|Pterostichus multipunctatus
35
GBCOU6148-14|Pterostichus multipunctatus
GBCOU6153-14|Pterostichus multipunctatus
GBCOU6170-14|Pterostichus multipunctatus
EUCAR742-11|Pterostichus multipunctatus
63
EUCAR746-11|Pterostichus multipunctatus
EUCAR747-11|Pterostichus multipunctatus
GBCOU4892-14|Pterostichus multipunctatus
89
GBCOU269-13|Pterostichus multipunctatus
54
GBMIX3818-19|Pterostichus multipunctatus
52
73
FBCOJ599-12|Pterostichus multipunctatus
GBMIX3819-19|Pterostichus multipunctatus
43
GBCOU9685-15|Pterostichus multipunctatus
72
EUCAR743-11|Pterostichus multipunctatus
96
EUCAR744-11|Pterostichus multipunctatus
EUCAR745-11|Pterostichus multipunctatus
60

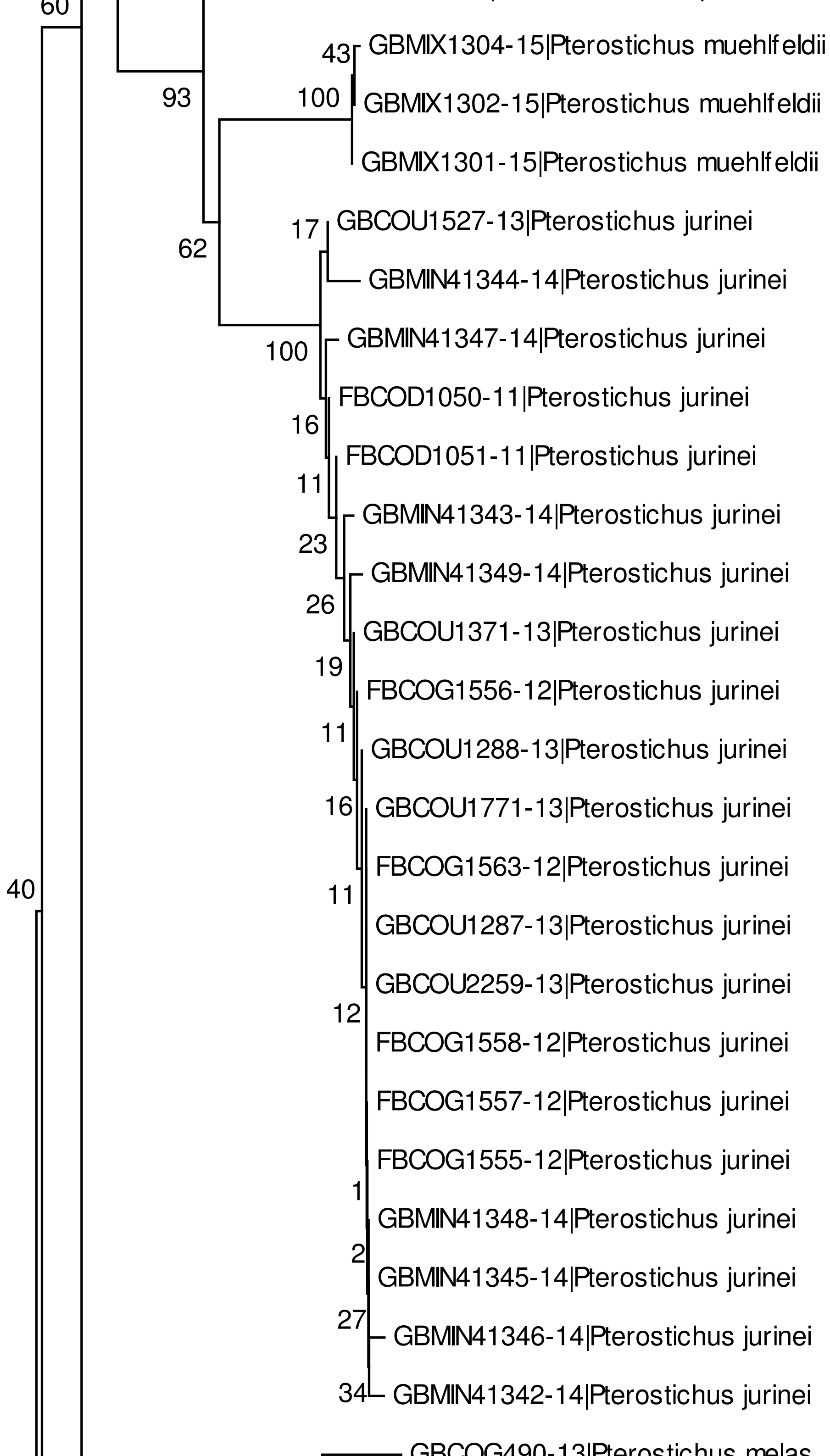
GBMIX1304-15|Pterostichus muehlfeldii
GBMIX1302-15|Pterostichus muehlfeldii
GBMIX1301-15|Pterostichus muehlfeldii
GBCOU1527-13|Pterostichus jurinei
GBMIN41344-14|Pterostichus jurinei
GBMIN41347-14|Pterostichus jurinei
FBCOD1050-11|Pterostichus jurinei
FBCOD1051-11|Pterostichus jurinei
GBMIN41343-14|Pterostichus jurinei
GBMIN41349-14|Pterostichus jurinei
GBCOU1371-13|Pterostichus jurinei
FBCOG1556-12|Pterostichus jurinei
GBCOU1288-13|Pterostichus jurinei
GBCOU1771-13|Pterostichus jurinei
FBCOG1563-12|Pterostichus jurinei
GBCOU1287-13|Pterostichus jurinei
GBCOU2259-13|Pterostichus jurinei
FBCOG1558-12|Pterostichus jurinei
FBCOG1557-12|Pterostichus jurinei
FBCOG1555-12|Pterostichus jurinei
GBMIN41348-14|Pterostichus jurinei
GBMIN41345-14|Pterostichus jurinei
GBMIN41346-14|Pterostichus jurinei
GBMIN41342-14|Pterostichus jurinei

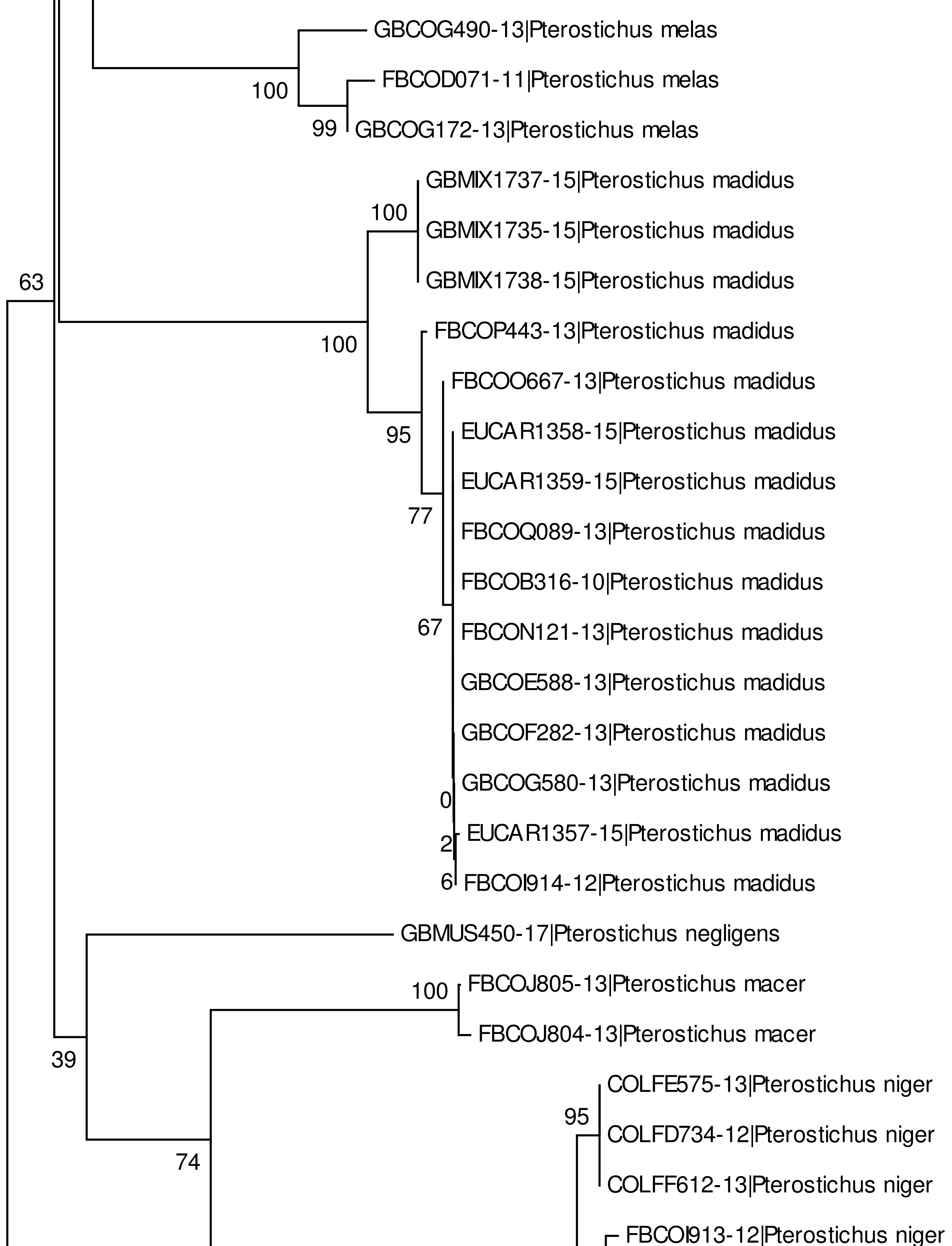
GBCOG490-13|Pterostichus melas
FBCOD071-11|Pterostichus melas
GBCOG172-13|Pterostichus melas
100
99
GBMIX1737-15|Pterostichus madidus
GBMIX1735-15|Pterostichus madidus
GBMIX1738-15|Pterostichus madidus
100
63
FBCOP443-13|Pterostichus madidus
100
FBCOO667-13|Pterostichus madidus
EUCAR1358-15|Pterostichus madidus
95
EUCAR1359-15|Pterostichus madidus
FBCOQ089-13|Pterostichus madidus
77
FBCOB316-10|Pterostichus madidus
FBCON121-13|Pterostichus madidus
67
GBCOE588-13|Pterostichus madidus
GBCOF282-13|Pterostichus madidus
GBCOG580-13|Pterostichus madidus
0
EUCAR1357-15|Pterostichus madidus
2
FBCOI914-12|Pterostichus madidus
6
GBMUS450-17|Pterostichus negligens
FBCOJ805-13|Pterostichus macer
100
FBCOJ804-13|Pterostichus macer
39
COLFE575-13|Pterostichus niger
95
COLFD734-12|Pterostichus niger
74
COLFF612-13|Pterostichus niger
FBCOI913-12|Pterostichus niger

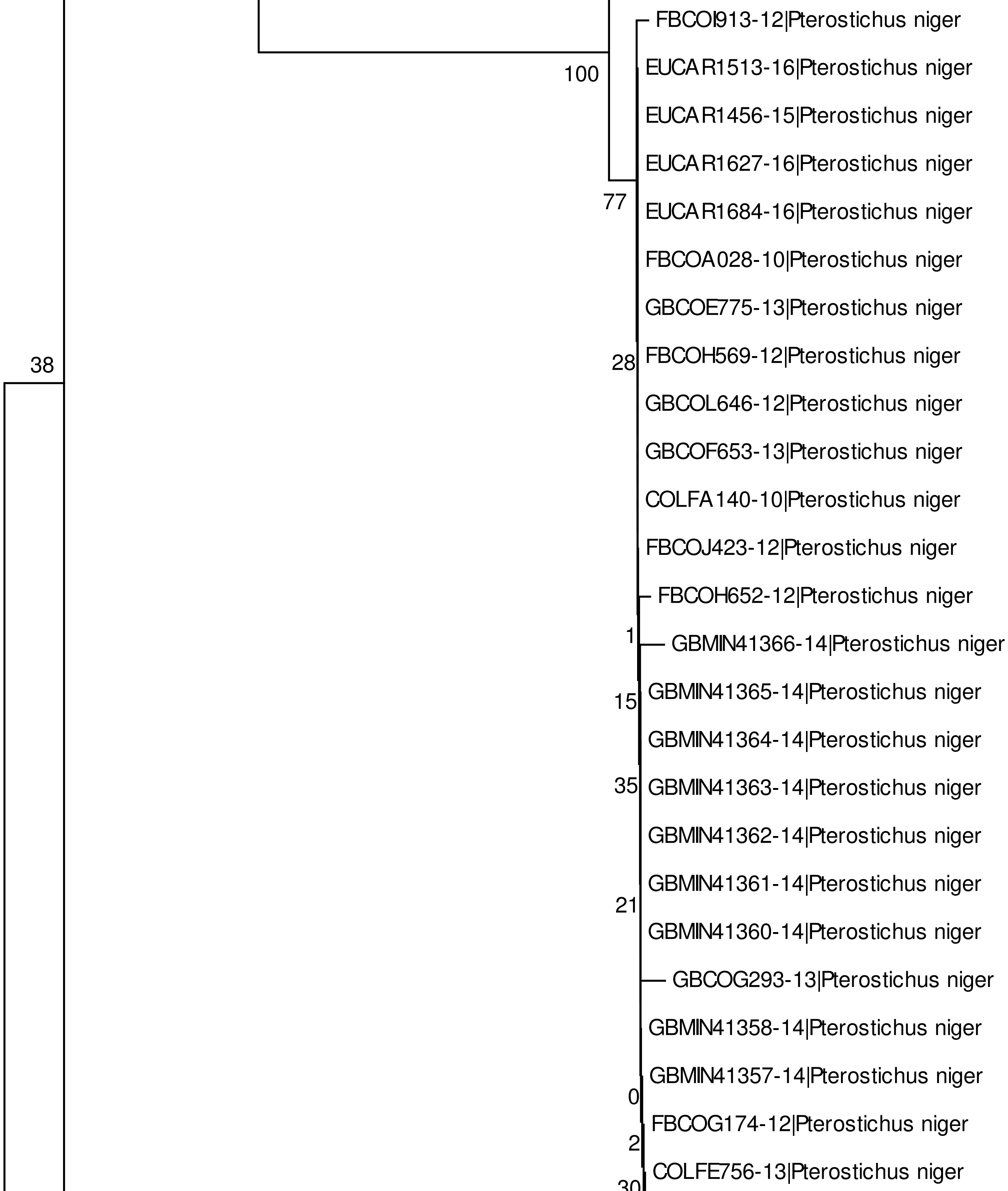
FBCOI913-12|Pterostichus niger
100
EUCAR1513-16|Pterostichus niger
EUCAR1456-15|Pterostichus niger
EUCAR1627-16|Pterostichus niger
77
EUCAR1684-16|Pterostichus niger
FBCOA028-10|Pterostichus niger
GBCOE775-13|Pterostichus niger
38
28
FBCOH569-12|Pterostichus niger
GBCOL646-12|Pterostichus niger
GBCOF653-13|Pterostichus niger
COLFA140-10|Pterostichus niger
FBCOJ423-12|Pterostichus niger
FBCOH652-12|Pterostichus niger
1
GBMIN41366-14|Pterostichus niger
GBMIN41365-14|Pterostichus niger
15
GBMIN41364-14|Pterostichus niger
35
GBMIN41363-14|Pterostichus niger
GBMIN41362-14|Pterostichus niger
GBMIN41361-14|Pterostichus niger
21
GBMIN41360-14|Pterostichus niger
GBCOG293-13|Pterostichus niger
GBMIN41358-14|Pterostichus niger
GBMIN41357-14|Pterostichus niger
0
FBCOG174-12|Pterostichus niger
2
COLFE756-13|Pterostichus niger

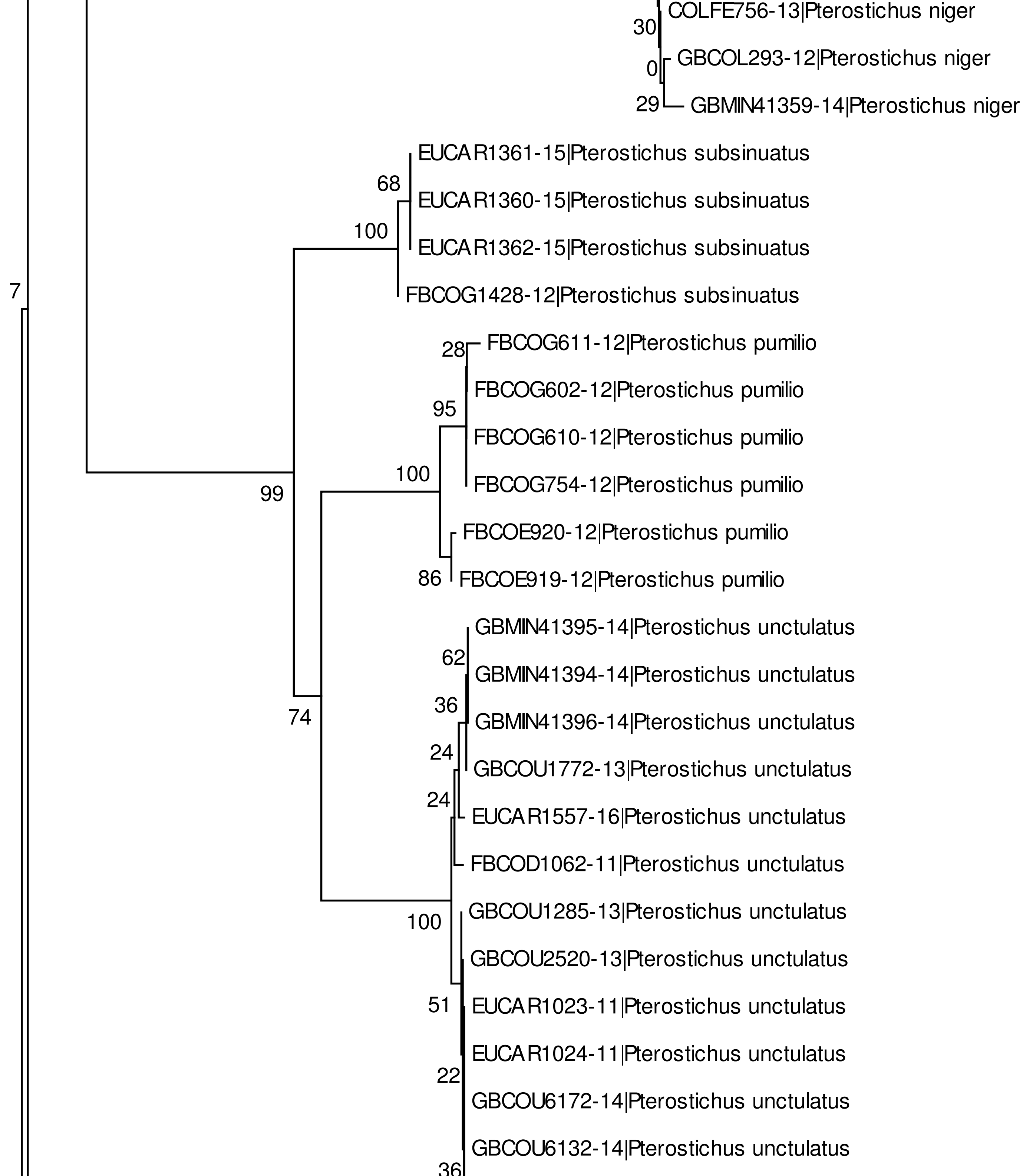
COLFE756-13|Pterostichus niger
30
GBCOL293-12|Pterostichus niger
0
29
GBMIN41359-14|Pterostichus niger
EUCAR1361-15|Pterostichus subsinuatus
68
EUCAR1360-15|Pterostichus subsinuatus
100
EUCAR1362-15|Pterostichus subsinuatus
7
FBCOG1428-12|Pterostichus subsinuatus
28
FBCOG611-12|Pterostichus pumilio
FBCOG602-12|Pterostichus pumilio
95
FBCOG610-12|Pterostichus pumilio
100
FBCOG754-12|Pterostichus pumilio
99
FBCOE920-12|Pterostichus pumilio
86
FBCOE919-12|Pterostichus pumilio
GBMIN41395-14|Pterostichus unctulatus
62
GBMIN41394-14|Pterostichus unctulatus
36
74
GBMIN41396-14|Pterostichus unctulatus
24
GBCOU1772-13|Pterostichus unctulatus
24
EUCAR1557-16|Pterostichus unctulatus
FBCOD1062-11|Pterostichus unctulatus
GBCOU1285-13|Pterostichus unctulatus
100
GBCOU2520-13|Pterostichus unctulatus
51
EUCAR1023-11|Pterostichus unctulatus
EUCAR1024-11|Pterostichus unctulatus
22
GBCOU6172-14|Pterostichus unctulatus
GBCOU6132-14|Pterostichus unctulatus
36

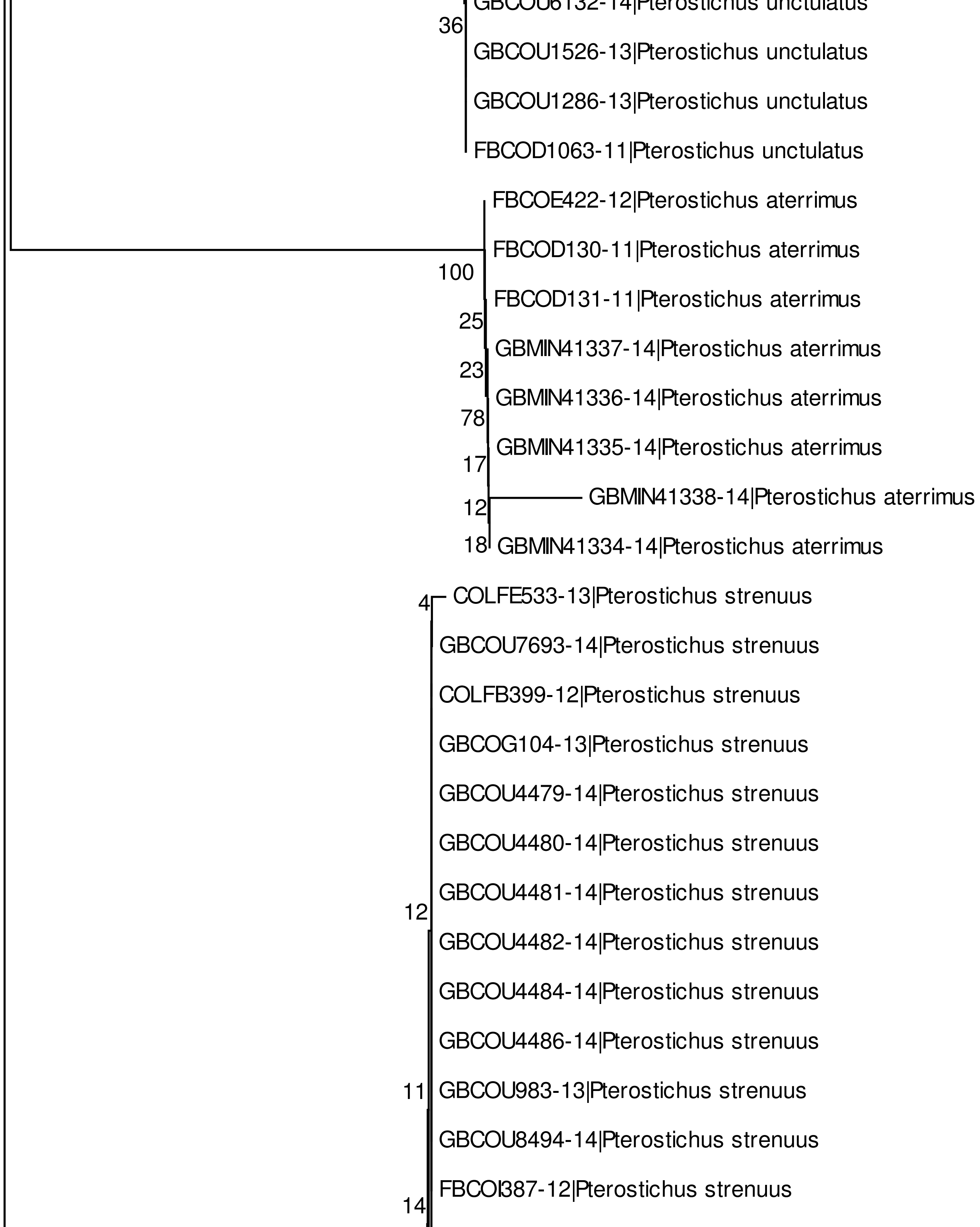
GBCOU6132-14|Pterostichus unctulatus
36
GBCOU1526-13|Pterostichus unctulatus
GBCOU1286-13|Pterostichus unctulatus
FBCOD1063-11|Pterostichus unctulatus
FBCOE422-12|Pterostichus aterrimus
FBCOD130-11|Pterostichus aterrimus
100
FBCOD131-11|Pterostichus aterrimus
25
GBMIN41337-14|Pterostichus aterrimus
23
GBMIN41336-14|Pterostichus aterrimus
78
GBMIN41335-14|Pterostichus aterrimus
17
GBMIN41338-14|Pterostichus aterrimus
12
18
GBMIN41334-14|Pterostichus aterrimus
4
COLFE533-13|Pterostichus strenuus
GBCOU7693-14|Pterostichus strenuus
COLFB399-12|Pterostichus strenuus
GBCOG104-13|Pterostichus strenuus
GBCOU4479-14|Pterostichus strenuus
GBCOU4480-14|Pterostichus strenuus
GBCOU4481-14|Pterostichus strenuus
12
GBCOU4482-14|Pterostichus strenuus
GBCOU4484-14|Pterostichus strenuus
GBCOU4486-14|Pterostichus strenuus
11
GBCOU983-13|Pterostichus strenuus
GBCOU8494-14|Pterostichus strenuus
FBCOI387-12|Pterostichus strenuus
14

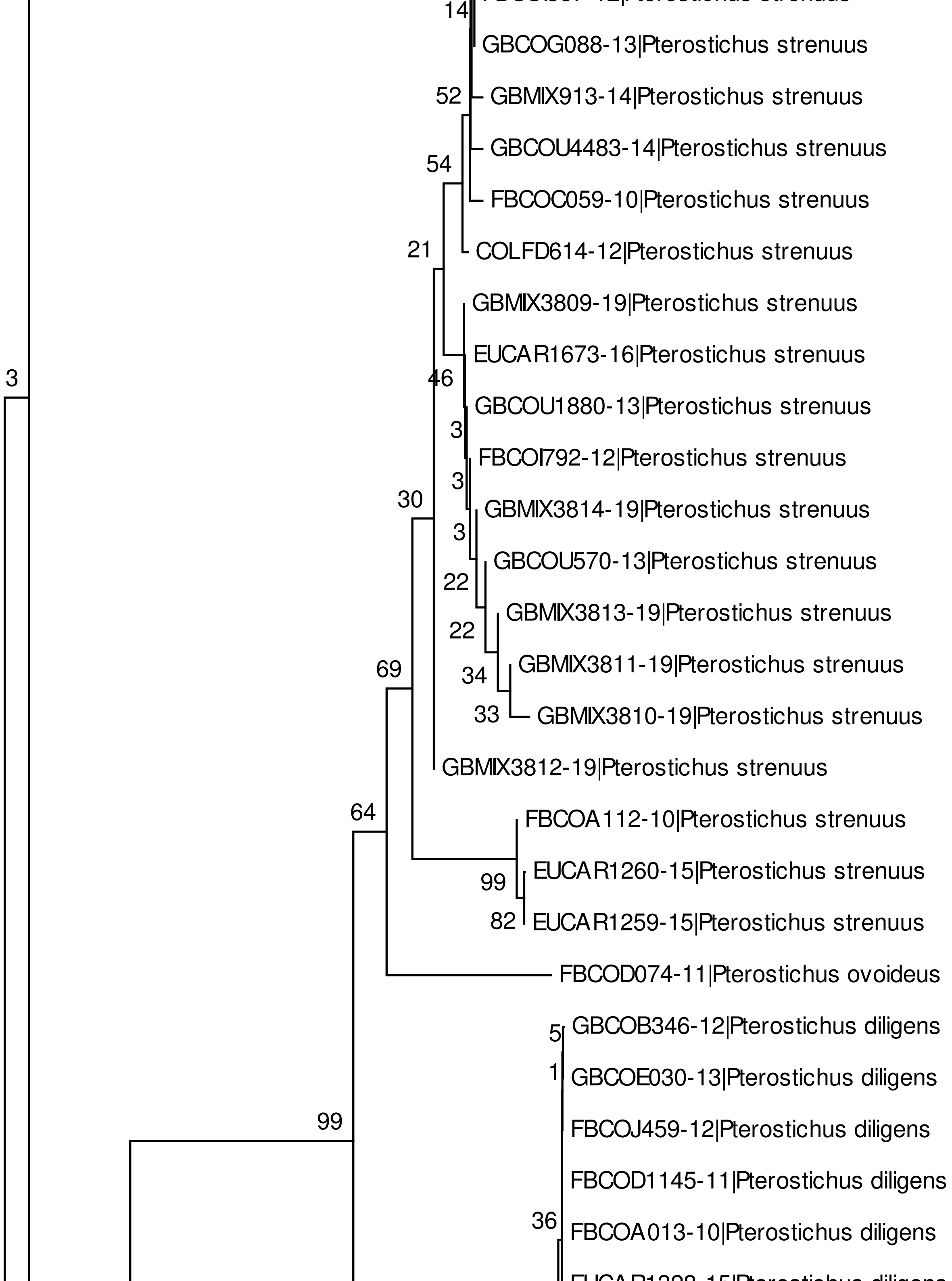
GBCOG088-13|Pterostichus strenuus
GBMIX913-14|Pterostichus strenuus
GBCOU4483-14|Pterostichus strenuus
FBCOC059-10|Pterostichus strenuus
COLFD614-12|Pterostichus strenuus
GBMIX3809-19|Pterostichus strenuus
EUCAR1673-16|Pterostichus strenuus
GBCOU1880-13|Pterostichus strenuus
FBCOI792-12|Pterostichus strenuus
GBMIX3814-19|Pterostichus strenuus
GBCOU570-13|Pterostichus strenuus
GBMIX3813-19|Pterostichus strenuus
GBMIX3811-19|Pterostichus strenuus
GBMIX3810-19|Pterostichus strenuus
GBMIX3812-19|Pterostichus strenuus
FBCOA112-10|Pterostichus strenuus
EUCAR1260-15|Pterostichus strenuus
EUCAR1259-15|Pterostichus strenuus
FBCOD074-11|Pterostichus ovoideus
GBCOB346-12|Pterostichus diligens
GBCOE030-13|Pterostichus diligens
FBCOJ459-12|Pterostichus diligens
FBCOD1145-11|Pterostichus diligens
FBCOA013-10|Pterostichus diligens

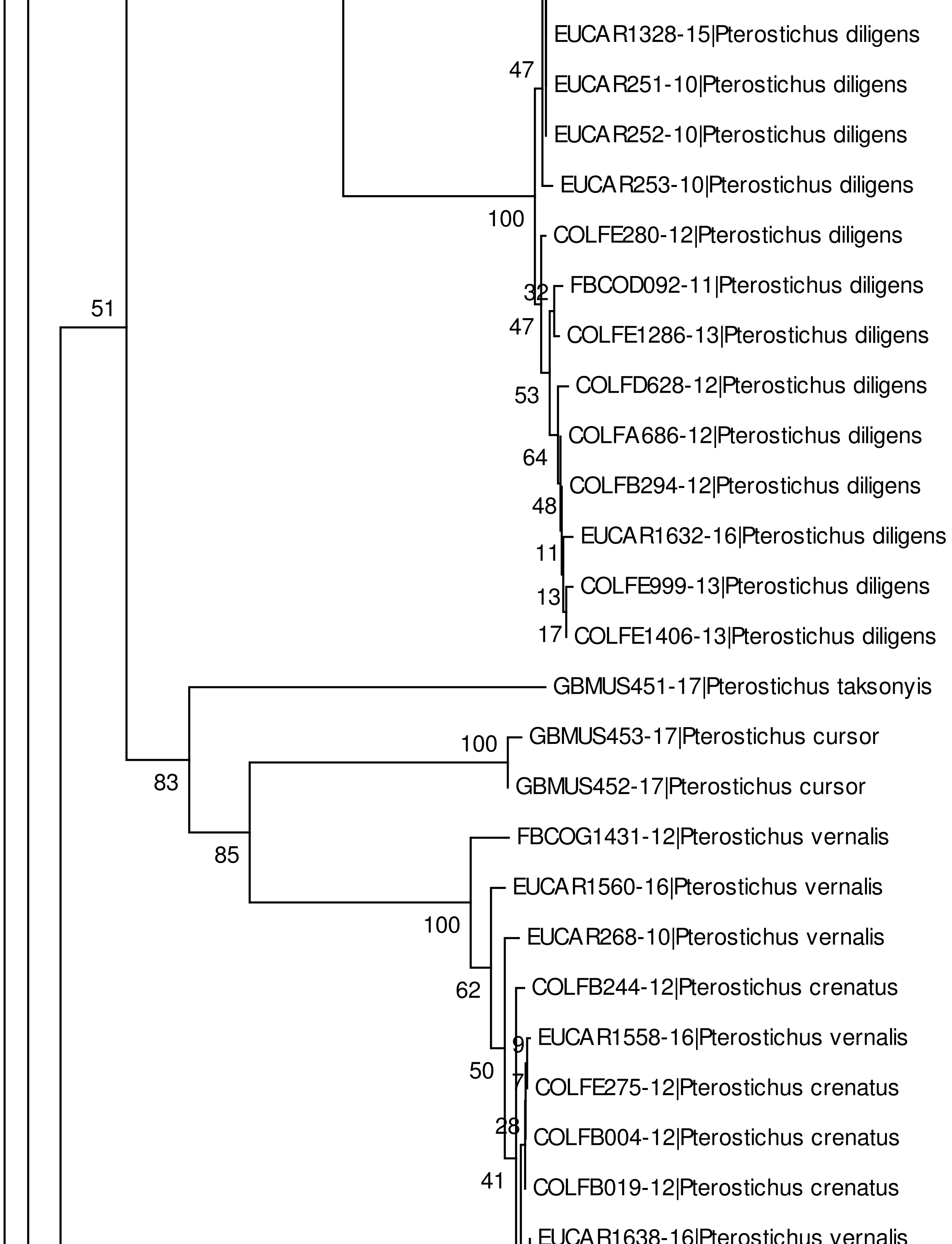
EUCAR1328-15|Pterostichus diligens
47
EUCAR251-10|Pterostichus diligens
EUCAR252-10|Pterostichus diligens
EUCAR253-10|Pterostichus diligens
100
COLFE280-12|Pterostichus diligens
32
FBCOD092-11|Pterostichus diligens
51
47
COLFE1286-13|Pterostichus diligens
53
COLFD628-12|Pterostichus diligens
COLFA686-12|Pterostichus diligens
64
COLFB294-12|Pterostichus diligens
48
EUCAR1632-16|Pterostichus diligens
11
COLFE999-13|Pterostichus diligens
13
17
COLFE1406-13|Pterostichus diligens
GBMUS451-17|Pterostichus taksonyis
100
GBMUS453-17|Pterostichus cursor
83
GBMUS452-17|Pterostichus cursor
FBCOG1431-12|Pterostichus vernalis
85
EUCAR1560-16|Pterostichus vernalis
100
EUCAR268-10|Pterostichus vernalis
62
COLFB244-12|Pterostichus crenatus
9
EUCAR1558-16|Pterostichus vernalis
50
7
COLFE275-12|Pterostichus crenatus
28
COLFB004-12|Pterostichus crenatus
41
COLFB019-12|Pterostichus crenatus
EUCAR1638-16|Pterostichus vernalis

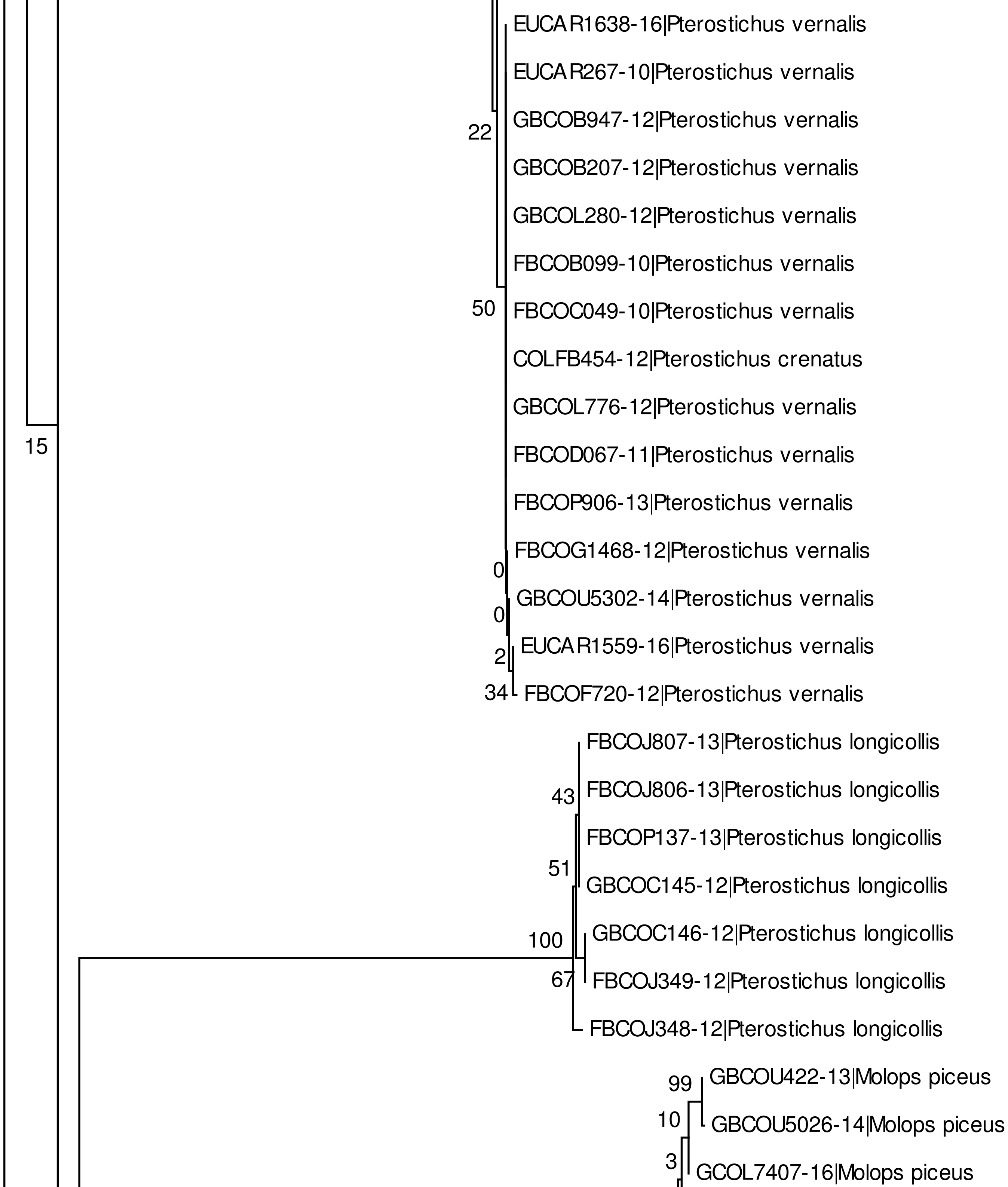
EUCAR1638-16|Pterostichus vernalis
EUCAR267-10|Pterostichus vernalis
22
GBCOB947-12|Pterostichus vernalis
GBCOB207-12|Pterostichus vernalis
GBCOL280-12|Pterostichus vernalis
FBCOB099-10|Pterostichus vernalis
50
FBCOC049-10|Pterostichus vernalis
COLFB454-12|Pterostichus crenatus
GBCOL776-12|Pterostichus vernalis
15
FBCOD067-11|Pterostichus vernalis
FBCOP906-13|Pterostichus vernalis
FBCOG1468-12|Pterostichus vernalis
0
GBCOU5302-14|Pterostichus vernalis
0
EUCAR1559-16|Pterostichus vernalis
2
34
FBCOF720-12|Pterostichus vernalis
FBCOJ807-13|Pterostichus longicollis
43
FBCOJ806-13|Pterostichus longicollis
FBCOP137-13|Pterostichus longicollis
51
GBCOC145-12|Pterostichus longicollis
100
GBCOC146-12|Pterostichus longicollis
67
FBCOJ349-12|Pterostichus longicollis
FBCOJ348-12|Pterostichus longicollis
99
GBCOU422-13|Molops piceus
10
GBCOU5026-14|Molops piceus
3
GCOL7407-16|Molops piceus

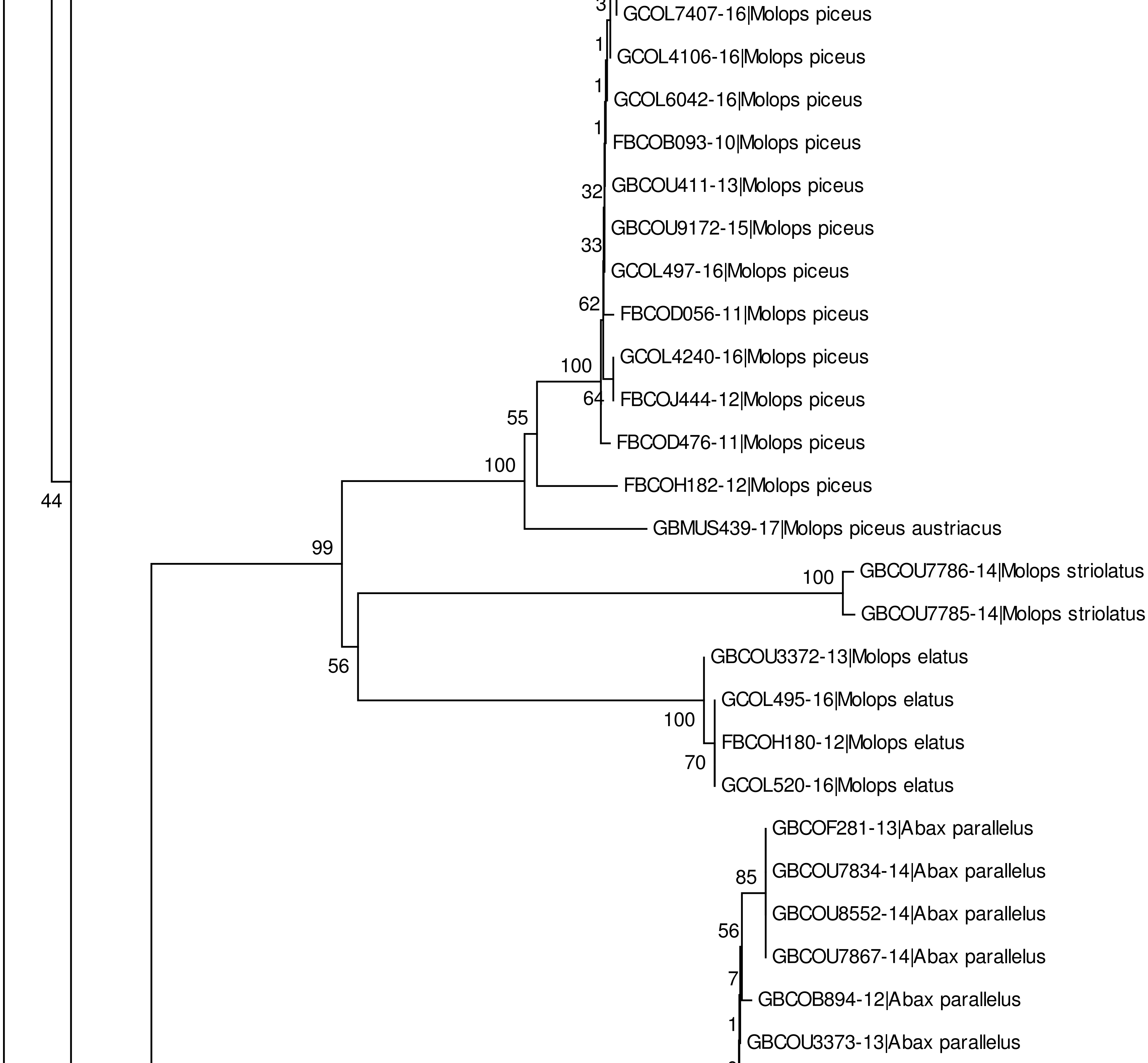
3
GCOL7407-16|Molops piceus
1
GCOL4106-16|Molops piceus
1
GCOL6042-16|Molops piceus
1
FBCOB093-10|Molops piceus
GBCOU411-13|Molops piceus
32
GBCOU9172-15|Molops piceus
33
GCOL497-16|Molops piceus
62
FBCOD056-11|Molops piceus
GCOL4240-16|Molops piceus
100
64
FBCOJ444-12|Molops piceus
55
FBCOD476-11|Molops piceus
100
FBCOH182-12|Molops piceus
44
GBMUS439-17|Molops piceus austriacus
99
100
GBCOU7786-14|Molops striolatus
GBCOU7785-14|Molops striolatus
56
GBCOU3372-13|Molops elatus
GCOL495-16|Molops elatus
100
FBCOH180-12|Molops elatus
70
GCOL520-16|Molops elatus
GBCOF281-13|Abax parallelus
GBCOU7834-14|Abax parallelus
85
GBCOU8552-14|Abax parallelus
56
GBCOU7867-14|Abax parallelus
7
GBCOB894-12|Abax parallelus
1
GBCOU3373-13|Abax parallelus

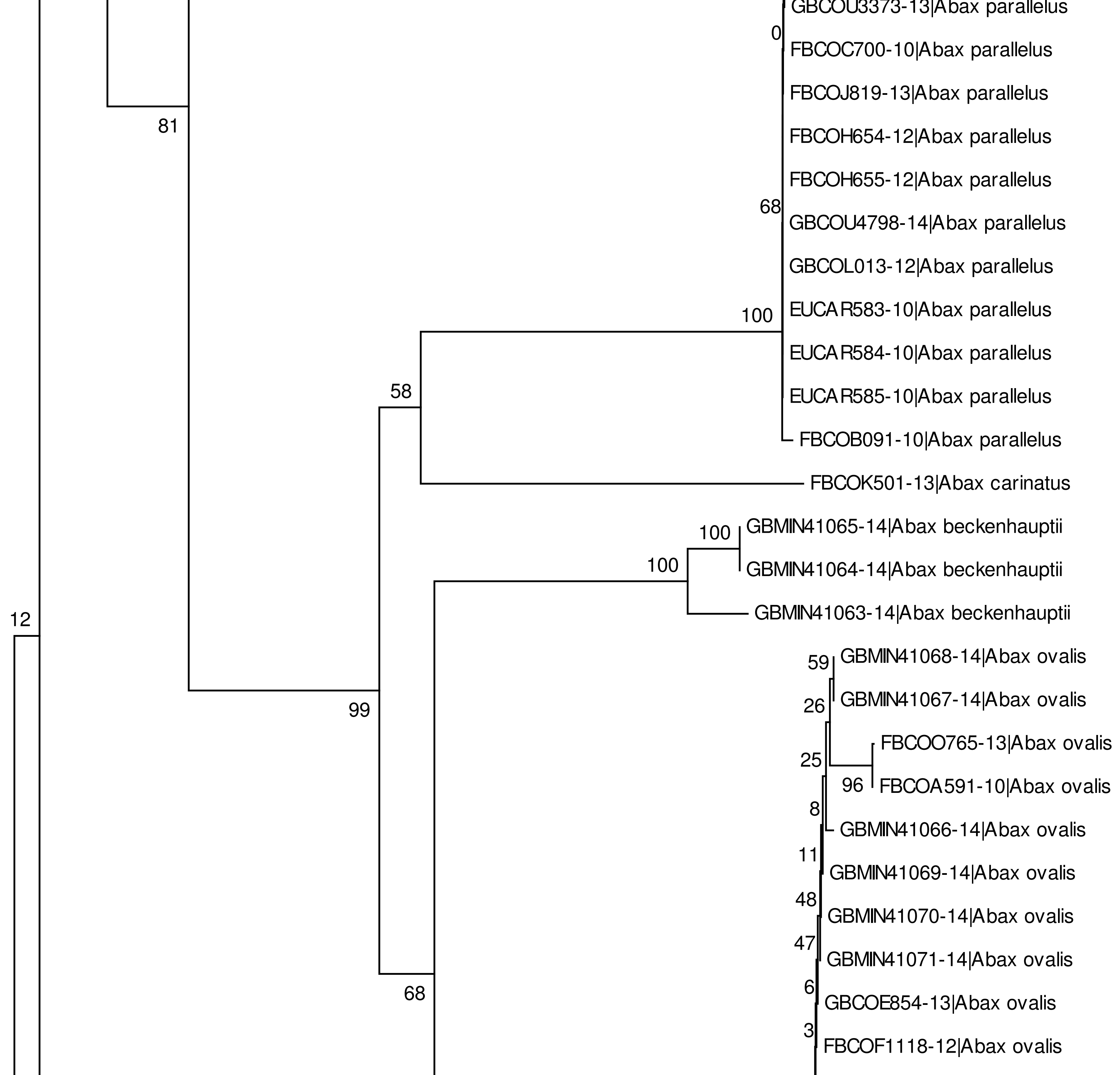

GBCOU3373-13|Abax parallelus
0
FBCOC700-10|Abax parallelus
FBCOJ819-13|Abax parallelus
FBCOH654-12|Abax parallelus
FBCOH655-12|Abax parallelus
68
GBCOU4798-14|Abax parallelus
GBCOL013-12|Abax parallelus
100
EUCAR583-10|Abax parallelus
EUCAR584-10|Abax parallelus
EUCAR585-10|Abax parallelus
FBCOB091-10|Abax parallelus
58
FBCOK501-13|Abax carinatus
81
100
GBMIN41065-14|Abax beckenhauptii
100
GBMIN41064-14|Abax beckenhauptii
GBMIN41063-14|Abax beckenhauptii
12
59
GBMIN41068-14|Abax ovalis
26
GBMIN41067-14|Abax ovalis
25
FBCOO765-13|Abax ovalis
96
FBCOA591-10|Abax ovalis
8
GBMIN41066-14|Abax ovalis
99
11
GBMIN41069-14|Abax ovalis
48
GBMIN41070-14|Abax ovalis
47
GBMIN41071-14|Abax ovalis
6
GBCOE854-13|Abax ovalis
68
3
FBCOF1118-12|Abax ovalis

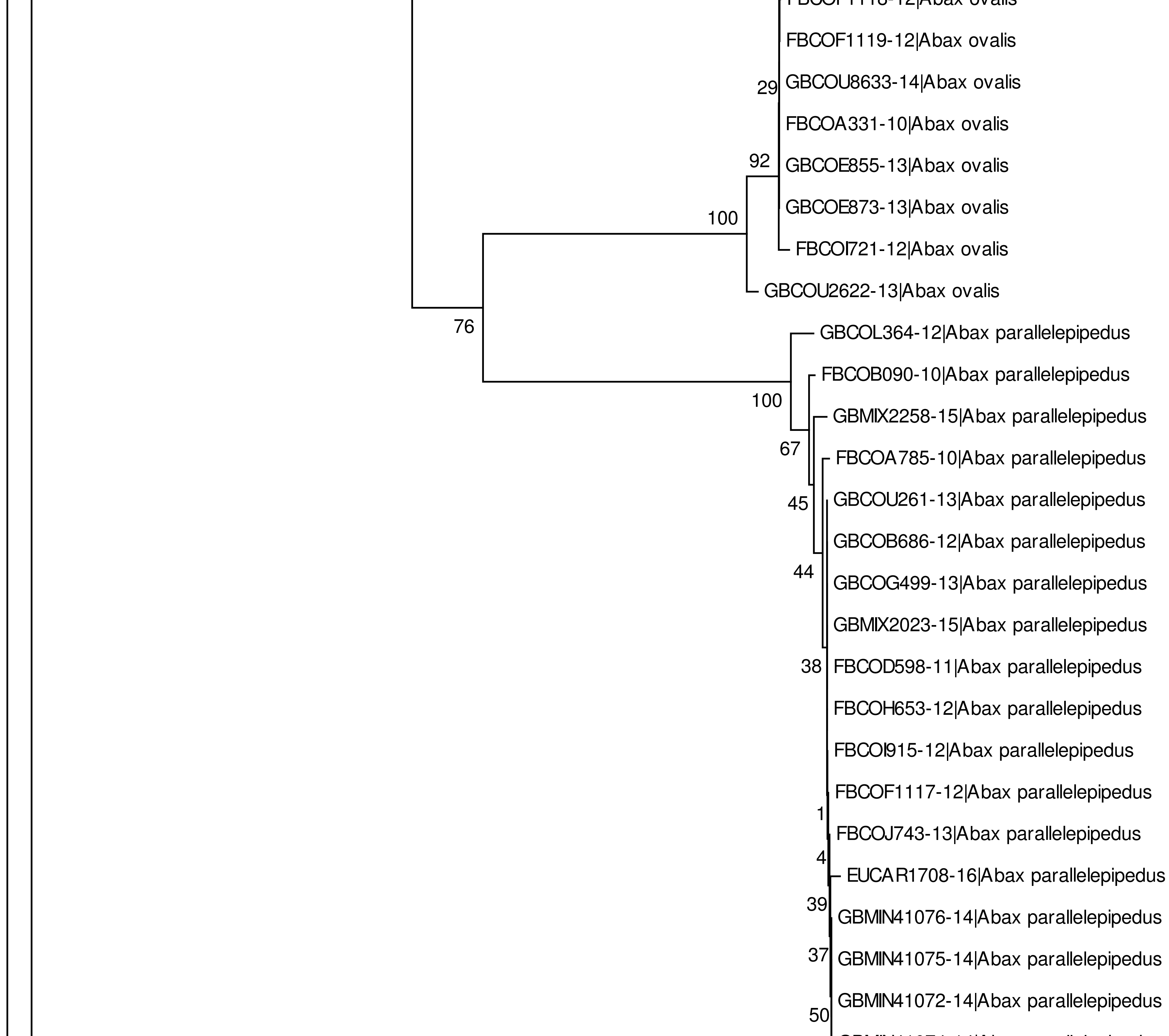
FBCOF1119-12|Abax ovalis
29
GBCOU8633-14|Abax ovalis
FBCOA331-10|Abax ovalis
92
GBCOE855-13|Abax ovalis
GBCOE873-13|Abax ovalis
100
FBCOI721-12|Abax ovalis
GBCOU2622-13|Abax ovalis
76
GBCOL364-12|Abax parallelepipedus
FBCOB090-10|Abax parallelepipedus
100
GBMIX2258-15|Abax parallelepipedus
67
FBCOA785-10|Abax parallelepipedus
45
GBCOU261-13|Abax parallelepipedus
GBCOB686-12|Abax parallelepipedus
44
GBCOG499-13|Abax parallelepipedus
GBMIX2023-15|Abax parallelepipedus
38
FBCOD598-11|Abax parallelepipedus
FBCOH653-12|Abax parallelepipedus
FBCOI915-12|Abax parallelepipedus
FBCOF1117-12|Abax parallelepipedus
1
FBCOJ743-13|Abax parallelepipedus
4
EUCAR1708-16|Abax parallelepipedus
39
GBMIN41076-14|Abax parallelepipedus
37
GBMIN41075-14|Abax parallelepipedus
GBMIN41072-14|Abax parallelepipedus
50

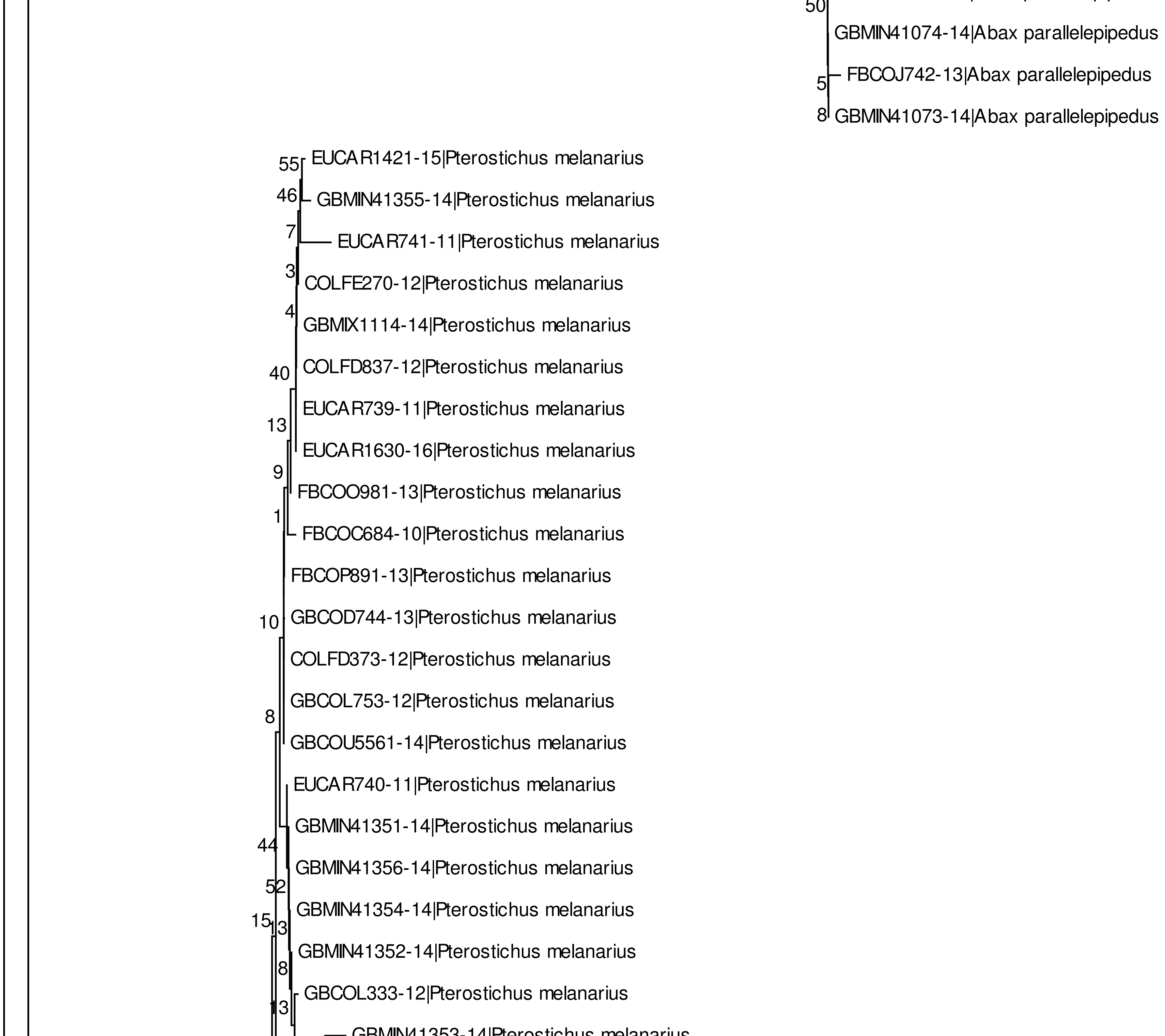
GBMIN41074-14|Abax parallelepipedus
FBCOJ742-13|Abax parallelepipedus
GBMIN41073-14|Abax parallelepipedus
EUCAR1421-15|Pterostichus melanarius
GBMIN41355-14|Pterostichus melanarius
EUCAR741-11|Pterostichus melanarius
COLFE270-12|Pterostichus melanarius
GBMIX1114-14|Pterostichus melanarius
COLFD837-12|Pterostichus melanarius
EUCAR739-11|Pterostichus melanarius
EUCAR1630-16|Pterostichus melanarius
FBCOO981-13|Pterostichus melanarius
FBCOC684-10|Pterostichus melanarius
FBCOP891-13|Pterostichus melanarius
GBCOD744-13|Pterostichus melanarius
COLFD373-12|Pterostichus melanarius
GBCOL753-12|Pterostichus melanarius
GBCOU5561-14|Pterostichus melanarius
EUCAR740-11|Pterostichus melanarius
GBMIN41351-14|Pterostichus melanarius
GBMIN41356-14|Pterostichus melanarius
GBMIN41354-14|Pterostichus melanarius
GBMIN41352-14|Pterostichus melanarius
GBCOL333-12|Pterostichus melanarius

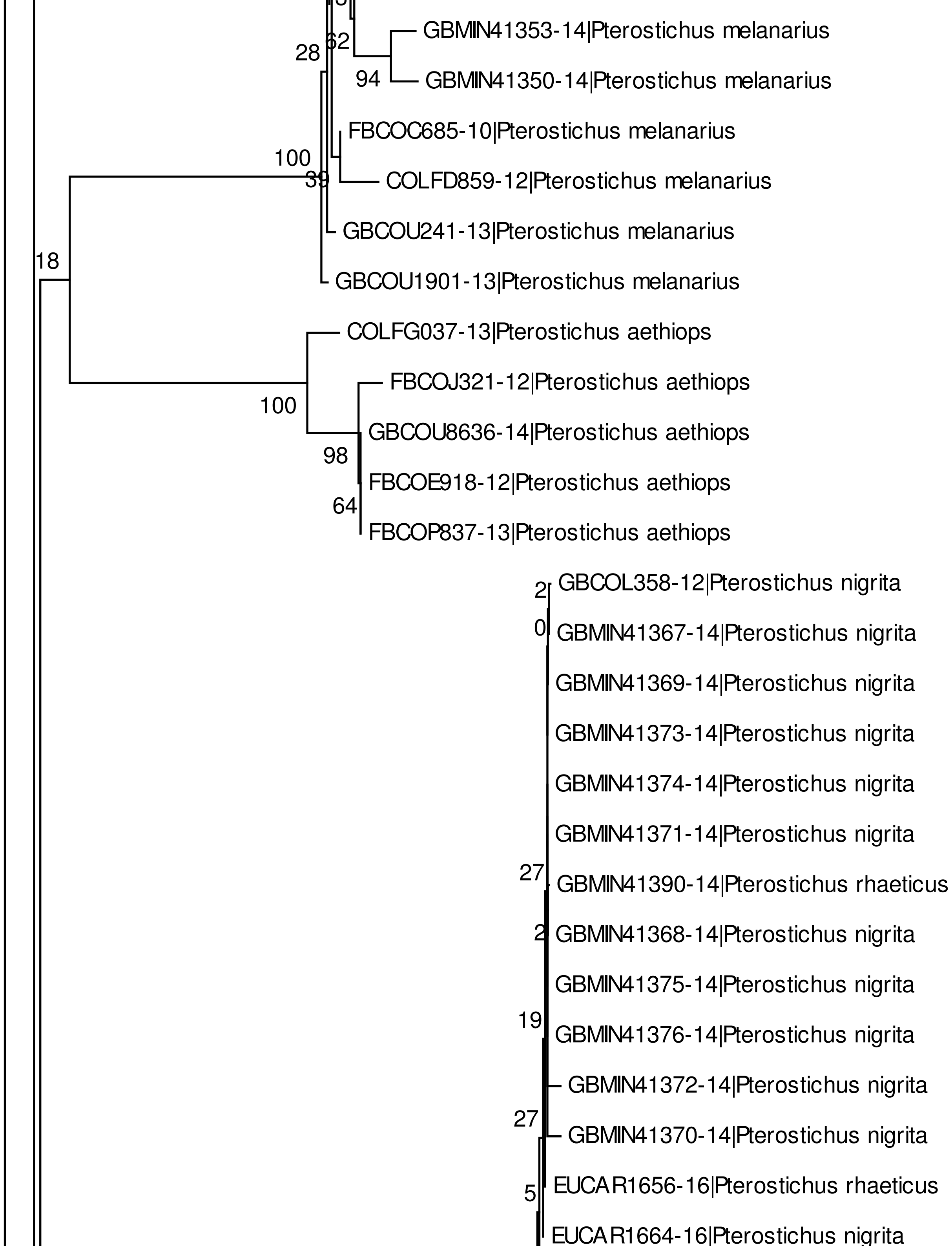

GBMIN41353-14|Pterostichus melanarius
GBMIN41350-14|Pterostichus melanarius
FBCOC685-10|Pterostichus melanarius
COLFD859-12|Pterostichus melanarius
GBCOU241-13|Pterostichus melanarius
GBCOU1901-13|Pterostichus melanarius
COLFG037-13|Pterostichus aethiops
FBCOJ321-12|Pterostichus aethiops
GBCOU8636-14|Pterostichus aethiops
FBCOE918-12|Pterostichus aethiops
FBCOP837-13|Pterostichus aethiops
GBCOL358-12|Pterostichus nigrita
GBMIN41367-14|Pterostichus nigrita
GBMIN41369-14|Pterostichus nigrita
GBMIN41373-14|Pterostichus nigrita
GBMIN41374-14|Pterostichus nigrita
GBMIN41371-14|Pterostichus nigrita
GBMIN41390-14|Pterostichus rhaeticus
GBMIN41368-14|Pterostichus nigrita
GBMIN41375-14|Pterostichus nigrita
GBMIN41376-14|Pterostichus nigrita
GBMIN41372-14|Pterostichus nigrita
GBMIN41370-14|Pterostichus nigrita
EUCAR1656-16|Pterostichus rhaeticus
EUCAR1664-16|Pterostichus nigrita
62
28
94
100
39
18
100
98
64
2
0
27
2
19
27
5

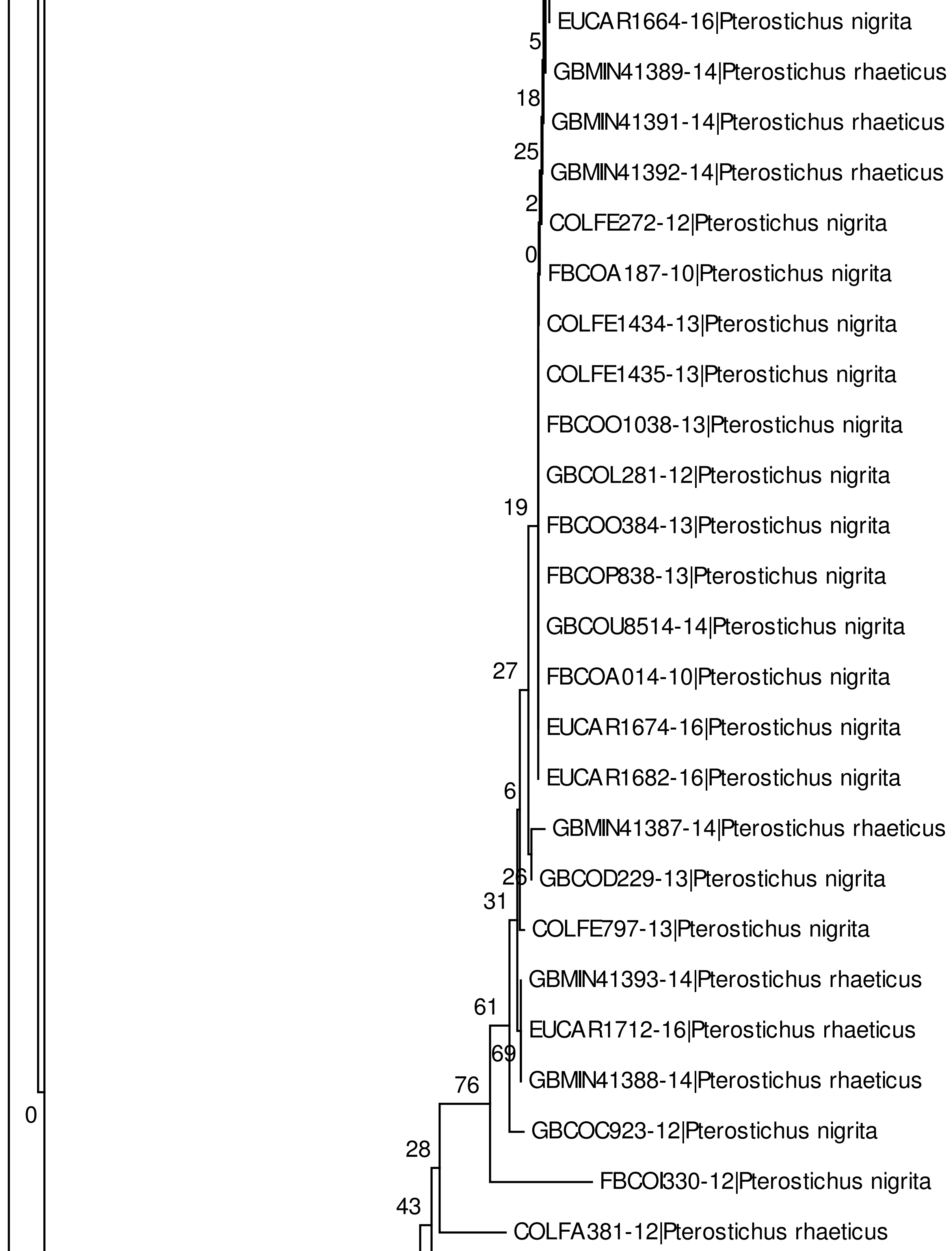
EUCAR1664-16|Pterostichus nigrita
5
GBMIN41389-14|Pterostichus rhaeticus
18
GBMIN41391-14|Pterostichus rhaeticus
25
GBMIN41392-14|Pterostichus rhaeticus
2
COLFE272-12|Pterostichus nigrita
0
FBCOA187-10|Pterostichus nigrita
COLFE1434-13|Pterostichus nigrita
COLFE1435-13|Pterostichus nigrita
FBCOO1038-13|Pterostichus nigrita
GBCOL281-12|Pterostichus nigrita
19
FBCOO384-13|Pterostichus nigrita
FBCOP838-13|Pterostichus nigrita
GBCOU8514-14|Pterostichus nigrita
27
FBCOA014-10|Pterostichus nigrita
EUCAR1674-16|Pterostichus nigrita
EUCAR1682-16|Pterostichus nigrita
6
GBMIN41387-14|Pterostichus rhaeticus
26
GBCOD229-13|Pterostichus nigrita
31
COLFE797-13|Pterostichus nigrita
GBMIN41393-14|Pterostichus rhaeticus
61
EUCAR1712-16|Pterostichus rhaeticus
69
GBMIN41388-14|Pterostichus rhaeticus
76
GBCOC923-12|Pterostichus nigrita
28
FBCOI330-12|Pterostichus nigrita
43
COLFA381-12|Pterostichus rhaeticus
0

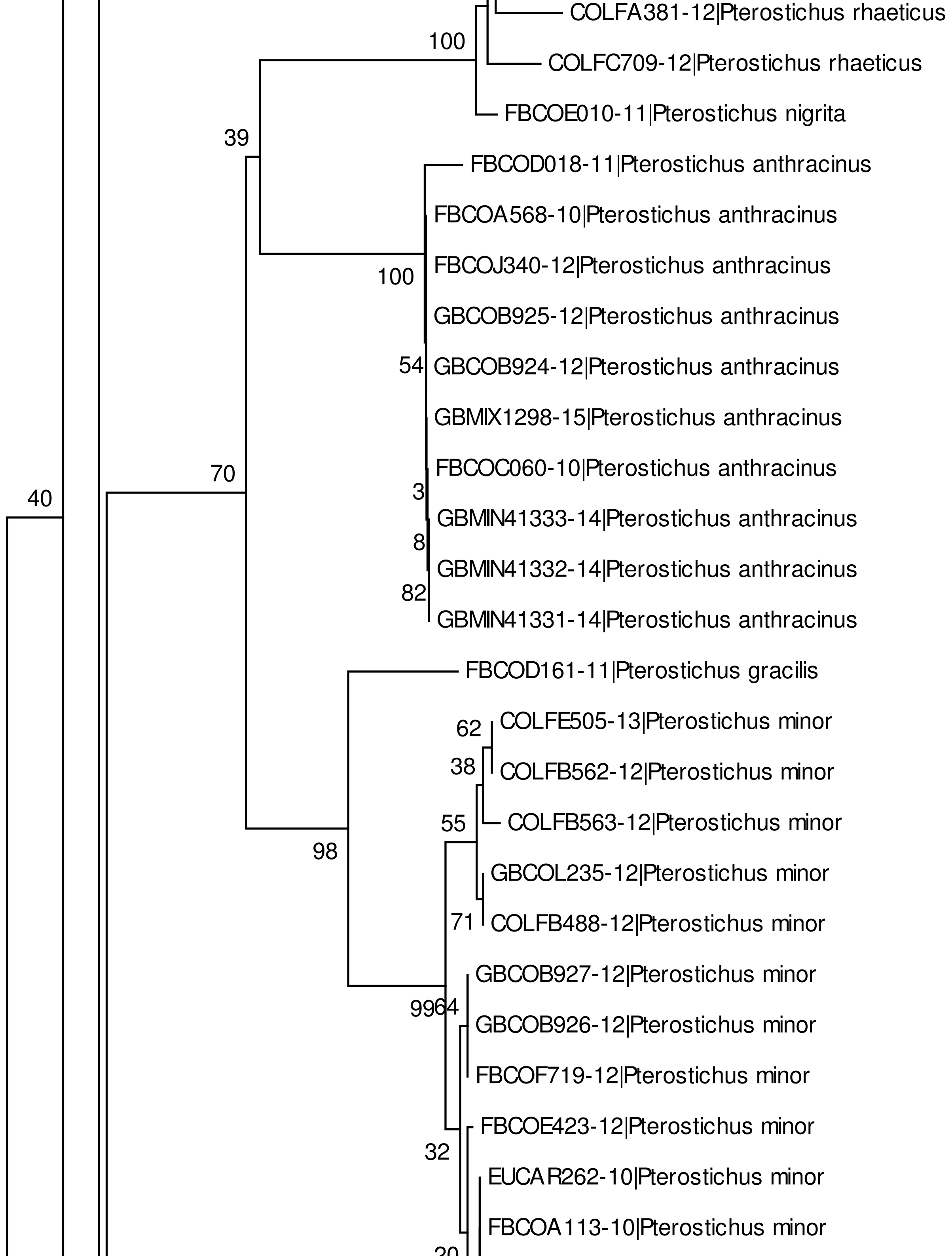
COLFA381-12|Pterostichus rhaeticus
100
COLFC709-12|Pterostichus rhaeticus
FBCOE010-11|Pterostichus nigrita
39
FBCOD018-11|Pterostichus anthracinus
FBCOA568-10|Pterostichus anthracinus
FBCOJ340-12|Pterostichus anthracinus
100
GBCOB925-12|Pterostichus anthracinus
54
GBCOB924-12|Pterostichus anthracinus
GBMIX1298-15|Pterostichus anthracinus
FBCOC060-10|Pterostichus anthracinus
70
40
3
GBMIN41333-14|Pterostichus anthracinus
8
GBMIN41332-14|Pterostichus anthracinus
82
GBMIN41331-14|Pterostichus anthracinus
FBCOD161-11|Pterostichus gracilis
62
COLFE505-13|Pterostichus minor
38
COLFB562-12|Pterostichus minor
55
COLFB563-12|Pterostichus minor
98
GBCOL235-12|Pterostichus minor
71
COLFB488-12|Pterostichus minor
GBCOB927-12|Pterostichus minor
9964
GBCOB926-12|Pterostichus minor
FBCOF719-12|Pterostichus minor
FBCOE423-12|Pterostichus minor
32
EUCAR262-10|Pterostichus minor
FBCOA113-10|Pterostichus minor

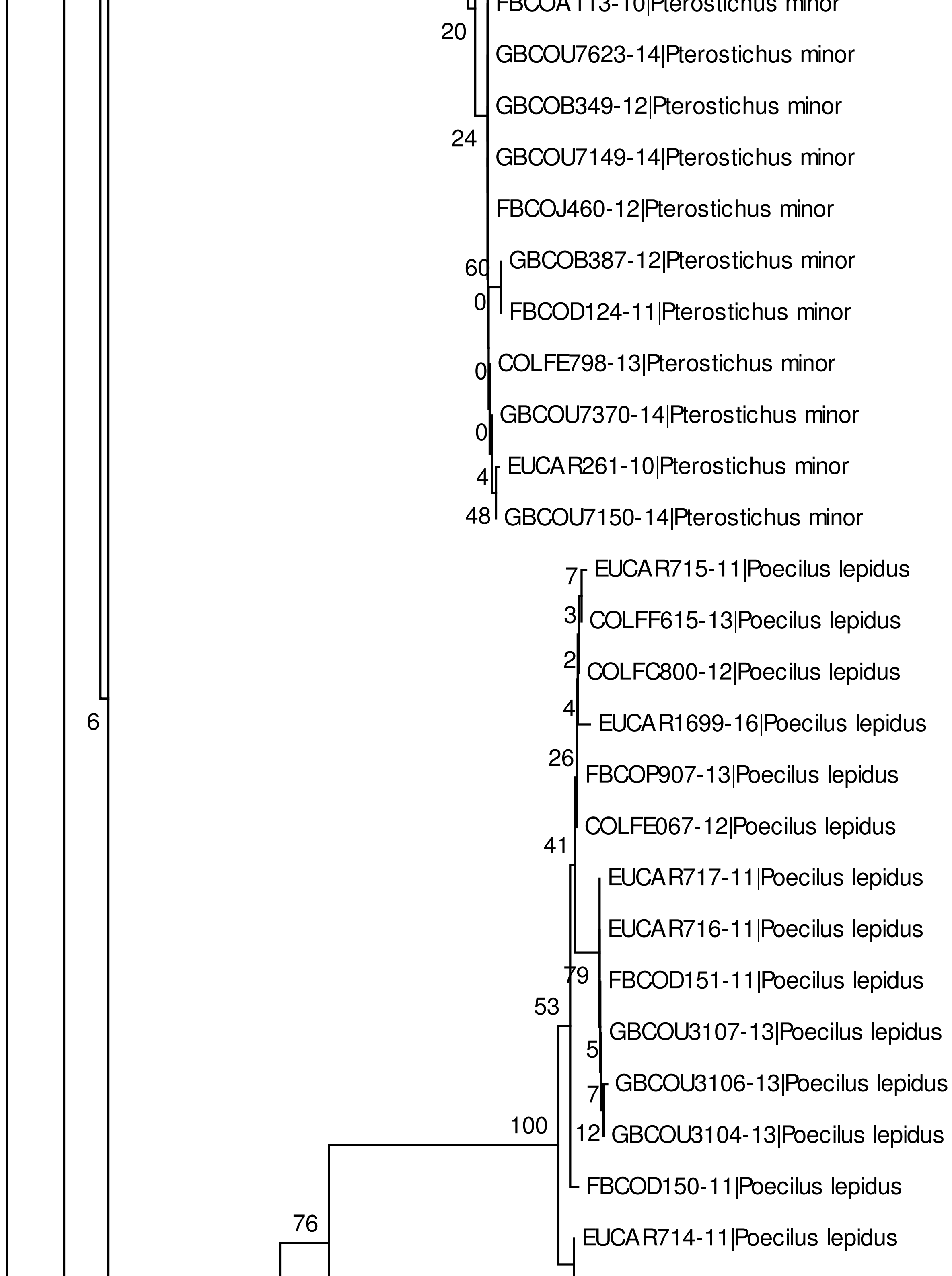
FBCOA113-10|Pterostichus minor
GBCOU7623-14|Pterostichus minor
GBCOB349-12|Pterostichus minor
GBCOU7149-14|Pterostichus minor
FBCOJ460-12|Pterostichus minor
GBCOB387-12|Pterostichus minor
FBCOD124-11|Pterostichus minor
COLFE798-13|Pterostichus minor
GBCOU7370-14|Pterostichus minor
EUCAR261-10|Pterostichus minor
GBCOU7150-14|Pterostichus minor
EUCAR715-11|Poecilus lepidus
COLFF615-13|Poecilus lepidus
COLFC800-12|Poecilus lepidus
EUCAR1699-16|Poecilus lepidus
FBCOP907-13|Poecilus lepidus
COLFE067-12|Poecilus lepidus
EUCAR717-11|Poecilus lepidus
EUCAR716-11|Poecilus lepidus
FBCOD151-11|Poecilus lepidus
GBCOU3107-13|Poecilus lepidus
GBCOU3106-13|Poecilus lepidus
GBCOU3104-13|Poecilus lepidus
FBCOD150-11|Poecilus lepidus
EUCAR714-11|Poecilus lepidus
20
24
60
0
0
0
4
48
7
3
2
4
26
41
79
53
5
7
12
100
76
6

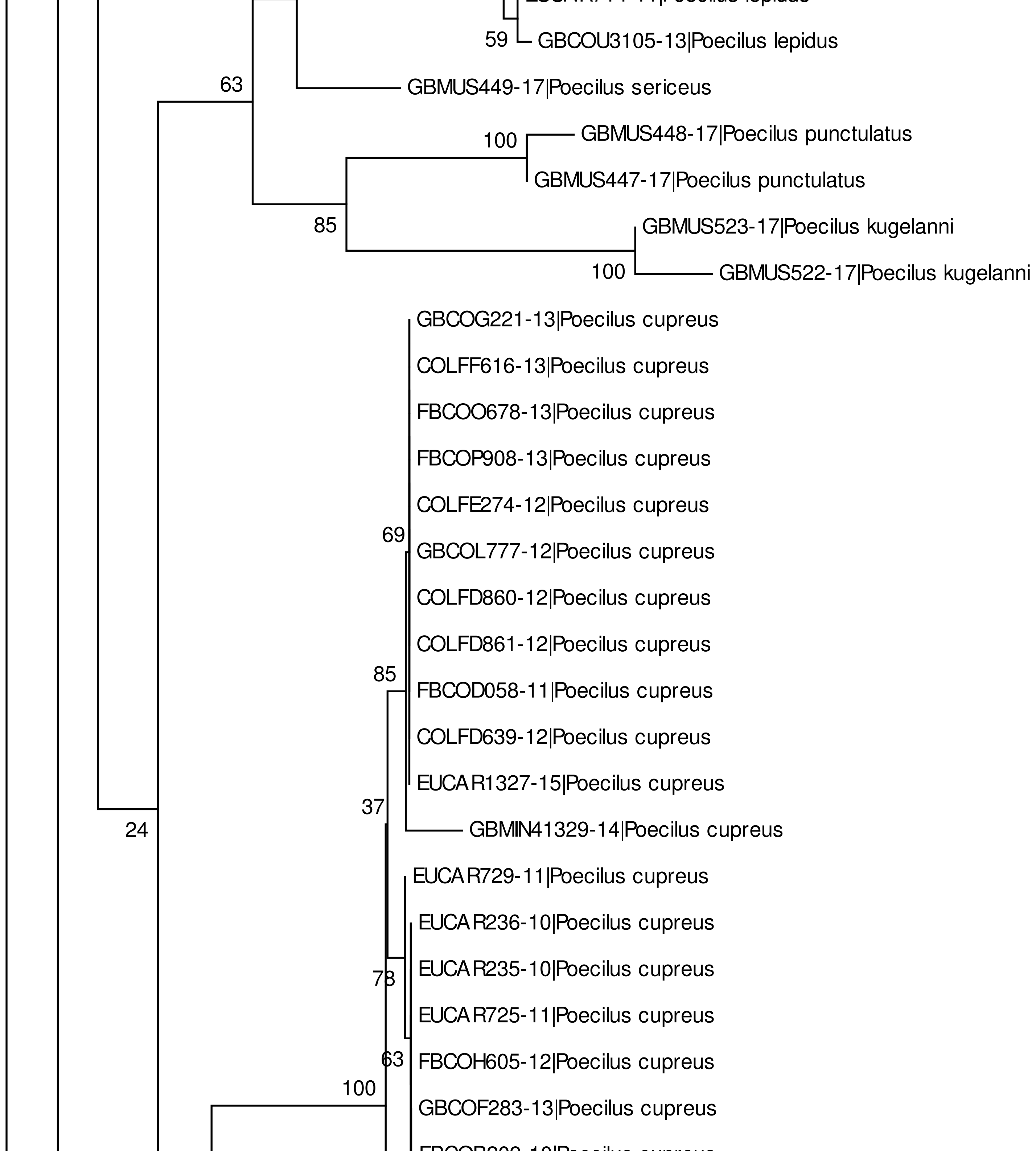

59
GBCOU3105-13|Poecilus lepidus
63
GBMUS449-17|Poecilus sericeus
100
GBMUS448-17|Poecilus punctulatus
GBMUS447-17|Poecilus punctulatus
85
GBMUS523-17|Poecilus kugelanni
100
GBMUS522-17|Poecilus kugelanni
GBCOG221-13|Poecilus cupreus
COLFF616-13|Poecilus cupreus
FBCOO678-13|Poecilus cupreus
FBCOP908-13|Poecilus cupreus
COLFE274-12|Poecilus cupreus
69
GBCOL777-12|Poecilus cupreus
COLFD860-12|Poecilus cupreus
COLFD861-12|Poecilus cupreus
85
FBCOD058-11|Poecilus cupreus
COLFD639-12|Poecilus cupreus
EUCAR1327-15|Poecilus cupreus
37
24
GBMIN41329-14|Poecilus cupreus
EUCAR729-11|Poecilus cupreus
EUCAR236-10|Poecilus cupreus
EUCAR235-10|Poecilus cupreus
78
EUCAR725-11|Poecilus cupreus
63
FBCOH605-12|Poecilus cupreus
100
GBCOF283-13|Poecilus cupreus

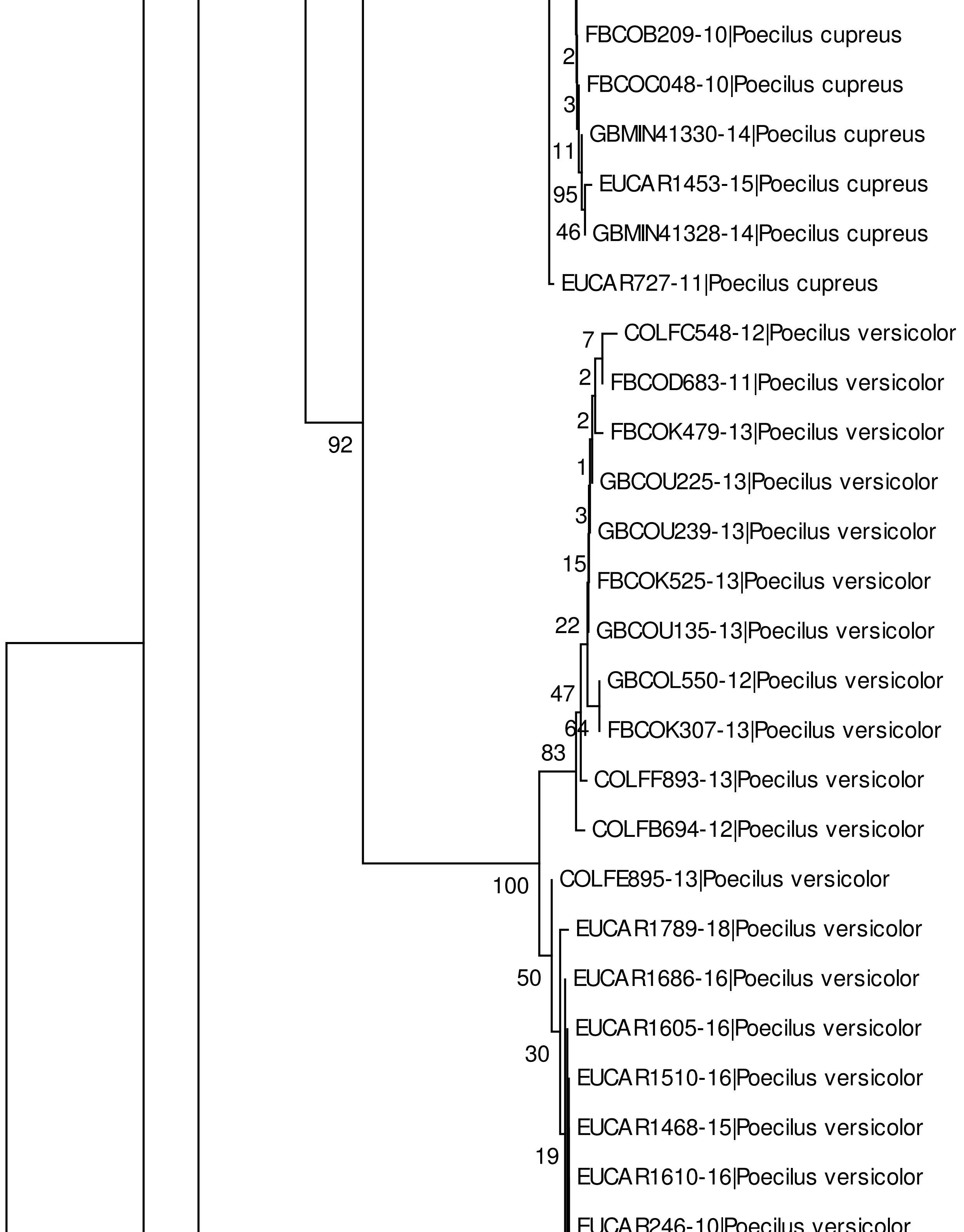
FBCOB209-10|Poecilus cupreus
2
FBCOC048-10|Poecilus cupreus
3
GBMIN41330-14|Poecilus cupreus
11
EUCAR1453-15|Poecilus cupreus
95
46
GBMIN41328-14|Poecilus cupreus
EUCAR727-11|Poecilus cupreus
7
COLFC548-12|Poecilus versicolor
2
FBCOD683-11|Poecilus versicolor
2
FBCOK479-13|Poecilus versicolor
92
1
GBCOU225-13|Poecilus versicolor
3
GBCOU239-13|Poecilus versicolor
15
FBCOK525-13|Poecilus versicolor
22
GBCOU135-13|Poecilus versicolor
GBCOL550-12|Poecilus versicolor
47
64
FBCOK307-13|Poecilus versicolor
83
COLFF893-13|Poecilus versicolor
COLFB694-12|Poecilus versicolor
100
COLFE895-13|Poecilus versicolor
EUCAR1789-18|Poecilus versicolor
50
EUCAR1686-16|Poecilus versicolor
EUCAR1605-16|Poecilus versicolor
30
EUCAR1510-16|Poecilus versicolor
EUCAR1468-15|Poecilus versicolor
19
EUCAR1610-16|Poecilus versicolor
EUCAR246-10|Poecilus versicolor

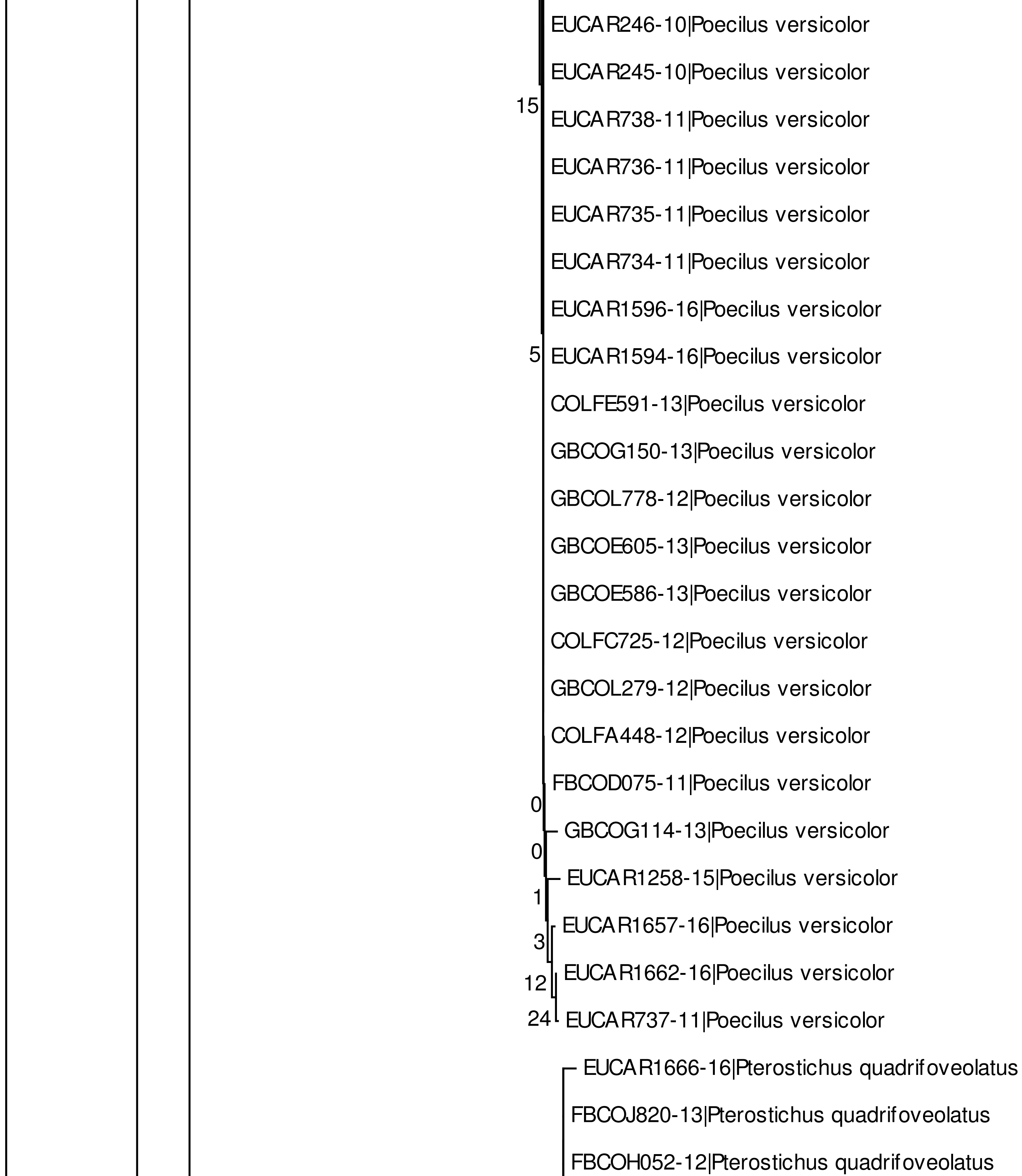
EUCAR246-10|Poecilus versicolor
EUCAR245-10|Poecilus versicolor
15
EUCAR738-11|Poecilus versicolor
EUCAR736-11|Poecilus versicolor
EUCAR735-11|Poecilus versicolor
EUCAR734-11|Poecilus versicolor
EUCAR1596-16|Poecilus versicolor
5
EUCAR1594-16|Poecilus versicolor
COLFE591-13|Poecilus versicolor
GBCOG150-13|Poecilus versicolor
GBCOL778-12|Poecilus versicolor
GBCOE605-13|Poecilus versicolor
GBCOE586-13|Poecilus versicolor
COLFC725-12|Poecilus versicolor
GBCOL279-12|Poecilus versicolor
COLFA448-12|Poecilus versicolor
FBCOD075-11|Poecilus versicolor
0
GBCOG114-13|Poecilus versicolor
0
EUCAR1258-15|Poecilus versicolor
1
EUCAR1657-16|Poecilus versicolor
3
EUCAR1662-16|Poecilus versicolor
12
24
EUCAR737-11|Poecilus versicolor
EUCAR1666-16|Pterostichus quadrifoveolatus
FBCOJ820-13|Pterostichus quadrifoveolatus
FBCOH052-12|Pterostichus quadrifoveolatus

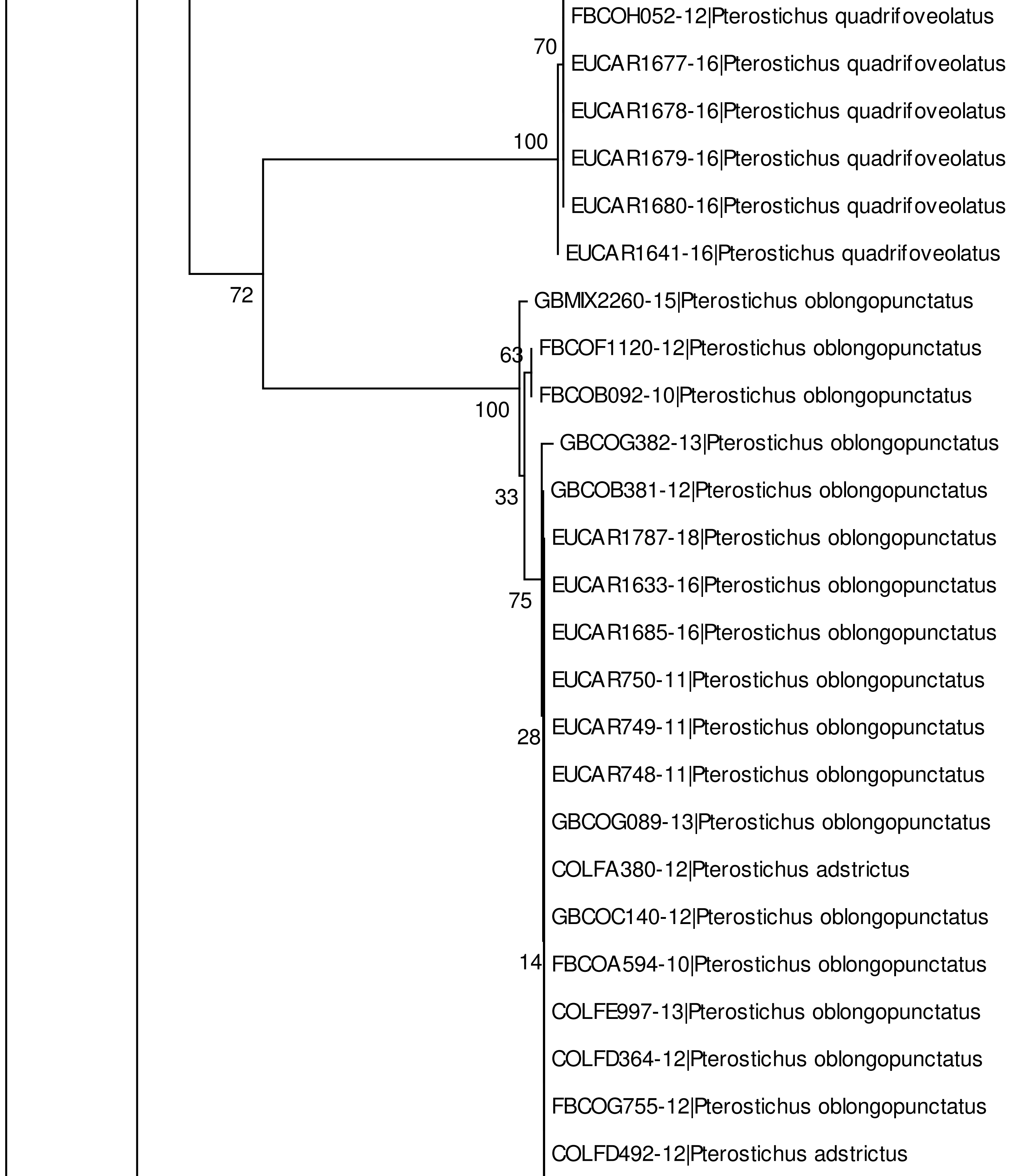

FBCOH052-12|Pterostichus quadrifoveolatus
70
EUCAR1677-16|Pterostichus quadrifoveolatus
EUCAR1678-16|Pterostichus quadrifoveolatus
100
EUCAR1679-16|Pterostichus quadrifoveolatus
EUCAR1680-16|Pterostichus quadrifoveolatus
EUCAR1641-16|Pterostichus quadrifoveolatus
72
GBMIX2260-15|Pterostichus oblongopunctatus
63
FBCOF1120-12|Pterostichus oblongopunctatus
FBCOB092-10|Pterostichus oblongopunctatus
100
GBCOG382-13|Pterostichus oblongopunctatus
33
GBCOB381-12|Pterostichus oblongopunctatus
EUCAR1787-18|Pterostichus oblongopunctatus
EUCAR1633-16|Pterostichus oblongopunctatus
75
EUCAR1685-16|Pterostichus oblongopunctatus
EUCAR750-11|Pterostichus oblongopunctatus
EUCAR749-11|Pterostichus oblongopunctatus
28
EUCAR748-11|Pterostichus oblongopunctatus
GBCOG089-13|Pterostichus oblongopunctatus
COLFA380-12|Pterostichus adstrictus
GBCOC140-12|Pterostichus oblongopunctatus
14
FBCOA594-10|Pterostichus oblongopunctatus
COLFE997-13|Pterostichus oblongopunctatus
COLFD364-12|Pterostichus oblongopunctatus
FBCOG755-12|Pterostichus oblongopunctatus
COLFD492-12|Pterostichus adstrictus

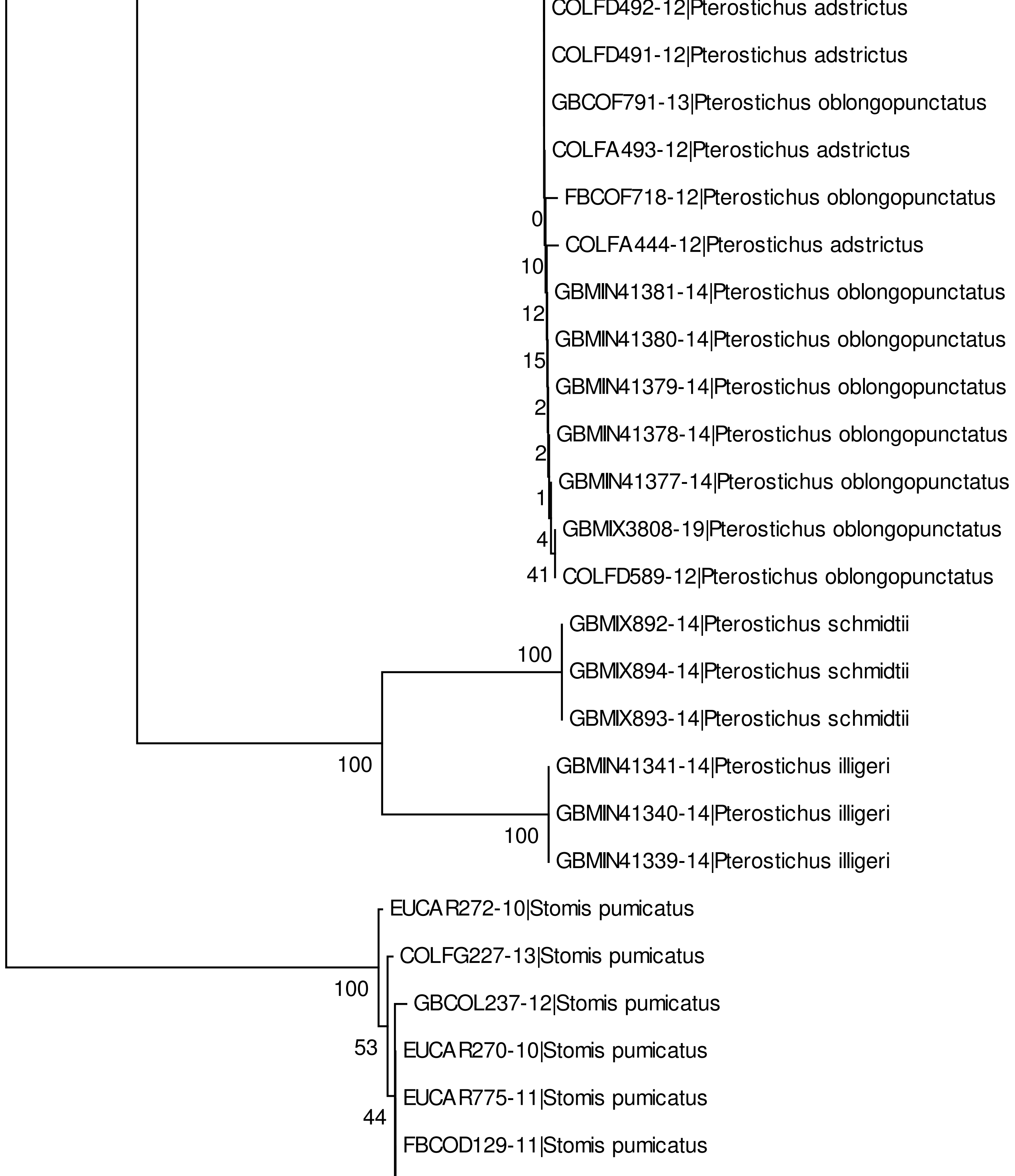
COLFD492-12|Pterostichus adstrictus
COLFD491-12|Pterostichus adstrictus
GBCOF791-13|Pterostichus oblongopunctatus
COLFA493-12|Pterostichus adstrictus
FBCOF718-12|Pterostichus oblongopunctatus
0
COLFA444-12|Pterostichus adstrictus
10
GBMIN41381-14|Pterostichus oblongopunctatus
12
GBMIN41380-14|Pterostichus oblongopunctatus
15
GBMIN41379-14|Pterostichus oblongopunctatus
2
GBMIN41378-14|Pterostichus oblongopunctatus
2
GBMIN41377-14|Pterostichus oblongopunctatus
1
GBMIX3808-19|Pterostichus oblongopunctatus
4
41
COLFD589-12|Pterostichus oblongopunctatus
GBMIX892-14|Pterostichus schmidtii
100
GBMIX894-14|Pterostichus schmidtii
GBMIX893-14|Pterostichus schmidtii
100
GBMIN41341-14|Pterostichus illigeri
GBMIN41340-14|Pterostichus illigeri
100
GBMIN41339-14|Pterostichus illigeri
EUCAR272-10|Stomis pumicatus
COLFG227-13|Stomis pumicatus
100
GBCOL237-12|Stomis pumicatus
53
EUCAR270-10|Stomis pumicatus
EUCAR775-11|Stomis pumicatus
44
FBCOD129-11|Stomis pumicatus

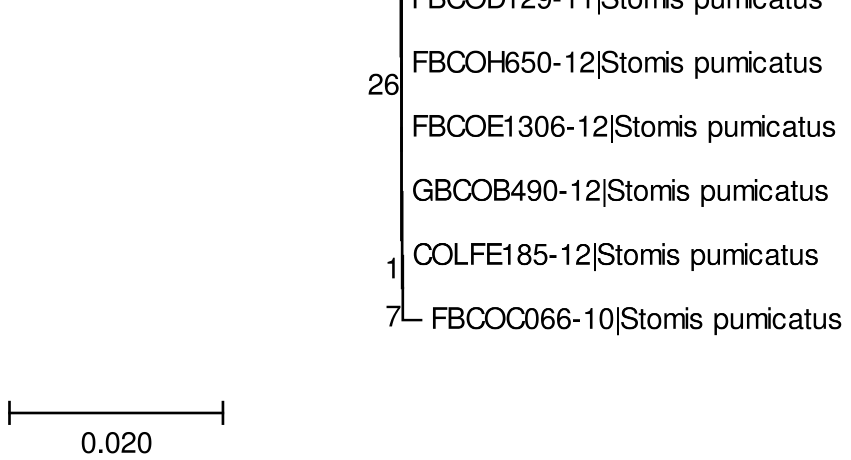
FBCOH650-12|Stomis pumicatus
26
FBCOE1306-12|Stomis pumicatus
GBCOB490-12|Stomis pumicatus
COLFE185-12|Stomis pumicatus
1
7
FBCOC066-10|Stomis pumicatus
0.020
